# Systematic evolution of *bZIP* transcription factors in Malvales and functional exploration of *AsbZIP14* and *AsbZIP41* in *Aquilaria sinensis*

**DOI:** 10.3389/fpls.2023.1243323

**Published:** 2023-08-30

**Authors:** Hao Zhang, Xupo Ding, Hao Wang, Huiqin Chen, Wenhua Dong, Jiahong Zhu, Jian Wang, Shiqing Peng, Haofu Dai, Wenli Mei

**Affiliations:** ^1^ Key Laboratory of Research and Development of Natural Product from Li Folk Medicine of Hainan Province, Institute of Tropical Bioscience and Biotechnology, Chinese Academy of Tropical Agricultural Sciences, Haikou, China; ^2^ Hainan Institute for Tropical Agricultural Resources, Chinese Academy of Tropical Agricultural Sciences, Haikou, China; ^3^ Key Laboratory of Germplasm Resources Biology of Tropical Special Ornamental Plants of Hainan, College of Forestry, Hainan University, Haikou, China

**Keywords:** *bZIP*, agarwood, Malvales, evolution, transcriptional regulation, chromone biosynthesis

## Abstract

**Introduction:**

Agarwood, the dark-brown resin produced by *Aquilaria* trees, has been widely used as incense, spice, perfume or traditional medicine and 2-(2-phenethyl) chromones (PECs) are the key markers responsible for agarwood formation. But the biosynthesis and regulatory mechanism of PECs were still not illuminated. The transcription factor of basic leucine zipper (bZIP) presented the pivotal regulatory roles in various secondary metabolites biosynthesis in plants, which might also contribute to regulate PECs biosynthesis. However, molecular evolution and function of bZIP are rarely reported in Malvales plants, especially in *Aquilaria* trees.

**Methods and results:**

Here, 1,150 *bZIPs* were comprehensively identified from twelve Malvales and model species genomes and the evolutionary process were subsequently analyzed. Duplication types and collinearity indicated that *bZIP* is an ancient or conserved TF family and recent whole genome duplication drove its evolution. Interesting is that fewer *bZIPs* in *A. sinensis* than that species also experienced two genome duplication events in Malvales. 62 AsbZIPs were divided into 13 subfamilies and gene structures, conservative domains, motifs, *cis*-elements, and nearby genes of *AsbZIPs* were further characterized. Seven *AsbZIPs* in subfamily D were significantly regulated by ethylene and agarwood inducer. As the typical representation of subfamily D, *AsbZIP14* and *AsbZIP41* were localized in nuclear and potentially regulated PECs biosynthesis by activating or suppressing type III polyketide synthases (PKSs) genes expression via interaction with the *AsPKS* promoters.

**Discussion:**

Our results provide a basis for molecular evolution of *bZIP* gene family in Malvales and facilitate the understanding the potential functions of *AsbZIP* in regulating 2-(2-phenethyl) chromone biosynthesis and agarwood formation.

## 1 Introduction

*Aquilaria sinensis* (Lour.) Spreng. is a special member of Malvales (Thymelaeaceae family) since it is one of the resource plants for agarwood production. This resin is a valuable aromatic ingredient which was used as perfumes and incense in religious rituals and ceremonies for centuries. Nowadays, agarwood is a highly demanded and indispensable ingredient in the perfume industry, and is welcomed as craft productions (Ding et al., 2020). In addition, it is also used as a traditional medicine in Chinese therapies and Ayurveda (Persoon, 2008; Naef, 2011; Ding et al., 2020). High-quality agarwood represents a precious, expensive, and scarce resource since healthy agarwood trees only produce few agarwood in nature unless they were affected by injury, such as burning, gnawing insects, lightning strikes, or fungal infection (Ding et al., 2020). Fungal inoculum is the most effective method for the artificial induction of agarwood production compared with chemical methods and physical methods (Sangareswari et al., 2016; Chhipa et al., 2017). This suggests that microbes play an important role in agarwood formation by priming agarwood tree immunity. Plants produce diverse secondary metabolites against biotic or abiotic stress. The previous research suggested that agarwood is a mixture of defense chemicals from agarwood trees in response to environmental stresses, especially stimulated by microbes pressures (Mohamed et al., 2010; Chhipa et al., 2017).

Terpenes and 2-(2-phenethyl) chromones (PECs) are the vital biological components and the main contributor to agarwood aroma. Previous research also indicated that chromones and their derivatives in the acetone extract from the high-quality agarwood occupied up to 60%, which implies that the contents of chromones and their derivatives might relate to the quality of agarwood (Ishihara et al., 1993). Type III polyketide synthases (PKSs) are pivotal for synthesizing the core flavonoids and their derivatives in the plant kingdom. They might also produce the precursors of chromone or its derivatives. However, its regulatory mechanism is unknown in *Aquilaria* trees or during agarwood formation. Transcription factors (TFs) play crucial roles in functional gene activation and regulation in model plants and crops. However, the role of TFs in chromones and agarwood formation has gained little attention (Liao et al., 2018; Wang et al., 2022d).

Transcription factors are one of the largest functional classes of proteins in eukaryotic genomes. They regulate nearly all biological processes including growth and development, hormone response, and environmental stress responses in plants. They are also essential in plant secondary metabolite accumulation according to their ability to regulate multiple functional genes in the metabolic pathways and cellular processes (Broun, 2004; Weirauch and Hughes, 2011). Recent studies suggest that TFs might contribute to the interaction between pathogen and *Aquilaria* trees to generate agarwood (Liu et al., 2022a). Basic leucine zipper proteins (bZIPs) are a ubiquitous family of plant transcription factors that share a basic region composed of a conserved DNA-contacting structure at the N-terminus and a characteristic and unique leucine zipper structure required for TF dimerization (Kouzarides and Ziff, 1989). Most plant *bZIPs* that prefer to recognize *cis* regulatory elements have ACGT core sequences, such as G-box (CACGTG), C-box (GACGTC), and A-box (TACGTA) (Foster et al., 1994; Dröge-Laser et al., 2018; Qu et al., 2022). The *bZIP* gene family (and their dimers thereof) perform a plethora of functions in plant physiological processes (Jakoby et al., 2002; Corrêa et al., 2008; Weirauch and Hughes, 2011).

Previous research identified 78 bZIPs that were sorted into 13 groups based on the phylogenetic tree of the bZIP basic region and predictor of conserved motifs in *Arabidopsis thaliana* (Dröge-Laser et al., 2018). The functional and regulatory processes of each AtbZIP subfamily were further summarized and highlighted according to extensive experimental data. For example, most AtbZIPs in group D were involved in plant hormone response and increasing plant tolerance for pathogen and xenobiotic stress in the environment (Fonseca et al., 2022). In addition, bZIP helps to regulate flavonoid pathways in *A. thaliana*, soybean, *Ginkgo biloba*, apple, or grapevine (Loyola et al., 2016; An et al., 2018; Job et al., 2018; Lian et al., 2018; Zhao et al., 2020). However, most research has focused on bZIPs of subfamily H (also called HY5) or subfamily G interacting with chalcone synthase (CHS) to regulate flavonoid accumulation. The involvement of bZIP in type III *PKS* expression or chromone content is rarely reported, especially for subgroup D of bZIPs.

The bZIP family of TFs is widely distributed in the plant kingdom and their subfamilies are analyzed in detail in various plants, especially the staple oil or food crops (wheat, rice, maize, Olive, soybean), the model plant (*Arabidopsis thaliana*) and in horticultural crops (litchi, pear, walnut, pomegranate, Chinese jujube, and sweet potato) (Wei et al., 2012; Zhang et al., 2014; Dröge-Laser et al., 2018; Zhang et al., 2018a; Agarwal et al., 2019; Yang et al., 2019; Rong et al., 2020; Zhang et al., 2020b; Ma et al., 2021; Hou et al., 2022; Wang et al., 2022c; Zhang et al., 2022b). Moreover, the *bZIP* gene families in medicinal plants such as *Carthamus tinctorius*, *Cannabis sativa*, *Andrographis paniculata*, *Isatis indigotica*, and *Salvia miltiorrhiza* were studied in recent years (Zhang et al., 2018b; Li et al., 2020; Guan et al., 2022; Jiang et al., 2022; Lu et al., 2022). Interestingly, bZIPs were also involved in mediated or increased the accumulation of effective ingredients in medicinal plants (Hao et al., 2019). However, the research was limited to the genome of individual species and comprehensive analysis of bZIPs in the same order has received little attention. The differentiation of gene family in the same orders might elucidate species evolution and trait formation, especially for the identification of special paralogous genes. Besides *A. sinensis*, Malvales contains numerous species that contribute to economic, cultural, or ecological levels, such as cotton (*Gossypium raimondii*), cocoa (*Theobroma cacao*), durian (*Durio zibethinus*), jute (*Corchorus capsularis* and *C. olitorius*). Therefore, a comprehensive analysis of bZIPs in *A. sinensis* and other Malvales plants will provide the evolutionary history and regulatory mechanism of bZIPs in the differentiation of species from Malvales.

This study aimed to identify and compare the *bZIP* gene family in the *A. sinensis* genome and eight other Malvales species genomes (*D. zibethinus*, *Hopea hainanensis*, *G. raimondii*, *T. cacao*, *C. capsularis*, *C. olitorius*, *Dipterocarpus turbinatus*, and *Hibiscus cannabinus*) to obtain a better picture of the size and evolution of the *bZIP* family in Malvales. Three outgroup genomes (*A. thaliana*, *Vitis vinifera*, and *Amborella trichopoda*) were used to reveal the evolutionary and differentiation process of the *bZIP* gene family in Malvales. Sixty-two *AsbZIP* genes in the *A. sinensis* genome were subsequently characterized to illustrate their phylogenetic relationship, conservative domains and motifs, gene structures, chromosomal location, duplication modes, collinearity, *cis*-elements, and sequence alignments between *A. sinensis* and ten other species. Furthermore, the expression profiles of genes from *bZIP* subfamily D were investigated by treating *A. sinensis* stems with ethylene and agarwood-inducer. Eventually, two *AsbZIPs* genes in subfamily D (*AsbZIP14* and *AsbZIP41*) that potentially participate in the regulation of chromone biosynthesis via activating or inhibiting the expression of *AsPKS* genes in *A. sinensis* were further investigated. These systematic results provide a theoretical basis for the further study of evolutionary processes of the *bZIP* gene family in Malvales and the roles of AsbZIP in agarwood formation and *Aquilaria* tree response to a wide range of environmental stresses.

## 2 Materials and methods

### 2.1 Plant material and databases

Five-year-old *A. sinensis* trees were collected from the Institute of Tropical Bioscience and Biotechnology (110° 19′ 246 E, 19° 59′ 756 N), Haikou, China. A 5 mm diameter and 1 cm-deep hole was drilled into the *A. sinensis* tree stem at a 45-degree downward angle from the trunk. Then, the needle of infusion was inserted into the hole and the agarwood inducer was slowly and continuously dripped into the xylem of the stems. The xylem tissues around the hole were taken (500 mg per sample) at 0, 3, 6, and 9 days after treatment. Ethylene (ET) treatment involved wrapping the stems of *A. sinensis* trees with absorbent cotton moistened with 1% ET and then intertwining them with plastic wrap. The treated tissues of stems were harvested (500 mg per sample) at 0, 3, 6, 12, 24, and 48 h after treatment. The collected samples were immediately frozen in liquid nitrogen and stored at -80°C in the laboratory prior to subsequent studies.

The genome files of *D. zibethinus*, *H. hainanensis*, *G. raimondii*, *T. cacao*, *C. capsularis*, *C. olitorius*, *D. turbinatus*, *H. cannabinus*, *V. vinifera*, *A. sinensis*, and *A. trichopoda* were downloaded from JGI (https://phytozome-next.jgi.doe.gov/) and NCBI (Argout et al., 2011; Project et al., 2013; Guan et al., 2014; Zhou et al., 2017; Wang et al., 2019; Ding et al., 2020; Zhang et al., 2020a; Zhang et al., 2021; Wang et al., 2022b; Zuccolo et al., 2011).

### 2.2 Identification of candidate *bZIP* genes from *A. sinensis* and eleven other species

The AsbZIPs in genomes were firstly identified by HMMER-3.1 with bZIP domains (PF00170 and PF12498) retrieving from the Pfam library (http://pfam.xfam.org). The homologous identification were used the seed sequences of all 78 AtbZIP proteins in *A. thaliana* downloaded from the TAIR library (https://www.arabidopsis.org/) to perform a blast search against the *A. sinensis* genome with e < 10^-5^ to obtain the protein sequences of AsbZIPs (Dröge-Laser et al., 2018). Then the non-redundant candidate database of AsbZIPs was obtained by combining the sequences from HMMER and homologous identification. The other elven non-redundant candidate databases were built by using the same method. Secondly, constructed the twelve non-redundant candidate databases as the final seed sequences and then aligned in each genome again to obtain the final candidates. These non-redundant candidates were subsequently screened by the NCBI-CDD library (https://www.ncbi.nlm.nih.gov/cdd), eggNOG-mapper library (http://eggnog-mapper.embl.de/), and Pfam database using the default parameters to verify the presence of conserved domains. The candidate proteins with no conserved domains of bZIP were removed and 62 genes were regarded as the final gene family of *bZIP* genes in *A*. *sinensis*.

### 2.3 Evolution and differentiation of *bZIP* genes in Marvales

The phylogenetic trees of species were initially constructed with r8s v 1.70 based on 302 single-copy genes from 12 species using OrthoFinder v 2.5.4. All bZIP protein sequences in 12 species were assembled as the final dataset for phylogenetic tree construction using FastTree v 2.1.11 after alignment with MAFFT v 7.310. The duplication and loss of *bZIP* genes in Marvales were analyzed using Notung v 2.9.13 based on two phylogenetic trees of species and bZIP protein sequences. The collinearity pairs of *bZIP* between *A. sinensis* and other ten species were detected using WGDI v 0.5.9 and visualized with JCVI v 0.8.4 (Goll et al., 2010). The protein alignments were translated into coding sequence alignments using an in-house Perl script of KaKs_calculator 3.0 for *Ks* (synonymous substitution rate) analysis. *Ka* (nonsynonymous substitution rate) and *Ks* values were then calculated based on the coding sequence alignments using the method of model averaging (MA) implemented in the KaKs_calculator 3.0 (Zhang, 2022). The divergence time was calculated with the formula *T = Ks/2r*. *Ks* being the synonymous substitutions per site and *r* represents the rate of divergence for nuclear genes from plants. The *r* was taken as 1.5×10^-8^ synonymous substitutions per site per year for dicotyledonous plants by evaluating chalcone synthase and alcohol dehydrogenase sequences using the fossil pollen data (Koch et al., 2000).

### 2.4 Bioinformatic analysis of *bZIP* genes in *A. sinensis*

The amino acid sequences of AsbZIP were submitted to the online software of ExPASy (https://www.expasy.org/) to obtain their isoelectric point (pI) and molecular weight (MW) and the subcellular localizations were predicted by WoLF PSORT software (https://wolfpsort.hgc.jp/) (Gasteiger et al., 2003). The MEME program (https://meme-suite.org/meme/tools/meme) was used to identify the conserved motifs of AsbZIP protein sequences and NCBI-CDD (https://www.ncbi.nlm.nih.gov/cdd) was used to collect information about the domain composition of AsbZIPs (Bailey et al., 2009). The results were visualized with CFVisual v 2.1 software (https://github.com/ChenHuilong1223/CFVisual). The exon/intron structure of *AsbZIP* genes was analyzed by the GSDS program (http://gsds.gao-lab.org/index.php) from the CDS and genomic sequence data (Hu et al., 2015). The *cis* acting elements of *AsbZIPs* were predicted from 2 kb DNA sequences upstream of the *AsbZIP* translation sites using the PlantCARE tool (http://bioinformatics.psb.ugent.be/webtools/plantcare/html/) (Lescot et al., 2002). Elements that respond to adversity stress or phytohormones were selected and visualized using CFVisual v 2.1 software. The initial prediction and analyses of *AsbZIP* genes and nearby genes were presented by the online software (https://www.chiplot.online/). Detailed information regarding the nearby genes was obtained from gene annotations (GFF3 format) of the *A. sinensis* genome. The protein sequences of these genes were submitted to the Eggnog-mapper library (http://eggnog-mapper.embl.de/) and agriGO (http://bioinfo.cau.edu.cn/agriGO/index.php) for gene classification and enrichment. The duplication events of the nearby genes were predicted by the script of duplicate_gene_classifer in MCscanX.

### 2.5 Multiple sequence alignment and phylogenetic analysis of AsbZIPs and AtbZIPs

The alignment of 140 full-length protein sequences (78 AtbZIPs and 62 AsbZIPs) was initially performed using MUSCLE with default parameters. A phylogenetic tree was constructed using the data with IQ-tree version 1.6.12 with the best model, VT+F+R7 (Minh et al., 2020). Analysis of the phylogenetic tree and the conserved motifs placed AsbZIPs into 13 groups (A-K, M and S) according to the classification method in *A. thaliana* and were visualized with Evolview 3.0 (http://www.evolgenius.info/) (Dröge-Laser et al., 2018).

### 2.6 Chromosome distribution and gene duplication of *AsbZIPs*

The information about the physical locations of *AsbZIP* genes in 8 chromosomes was obtained from the annotations of 62 genes (GFF3 format) and the *AsbZIP* genes were renamed according to their chromosomal locations. The duplication types of *bZIP1* genes in *A*. *sinensis* were analyzed by the script of the duplicate_gene_classifer in MCscanX and visualized by Circos v0.69 (Krzywinski et al., 2009). Collinearity analysis of As*bZIP* was also detected using WGDI v 0.5.9 (Sun et al., 2022b).

### 2.7 Transcriptome and quantitative real-time PCR (qRT-PCR) analysis

The expression levels of 62 *AsbZIP* genes were obtained from previous transcriptome data and the log2 fragments per kilobase per million mapped reads (FPKM) values of *AsbZIP* genes were visualized by Heml 1.0 software (Ding et al., 2020; Li et al., 2021a). Total RNA was isolated from collected samples with the RNAprep Pure Plant Plus kit (Tiangen, China) and stored as previously described. First-strand cDNA was immediately synthesized using a FastKing gDNA Dispelling RT SuperMix kit (Tiangen, China) and was employed as the template in quantitative real-time PCR (RT-qPCR). Meanwhile, all seven genes in subfamily D were selected for RT-qPCR validation analysis and histone was used as a housekeeping gene (Kumeta and Ito, 2010). Primers were designed by the IDT PrimerQuest tool (https://sg.idtdna.com/pages) (**Table S1**). Quantitative real-time PCR was conducted using SuperReal PreMix Plus (SYBR Green) (Tiangen, China) with the CFX96™ Real-Time system (Bio-Rad, Hercules, CA, USA) using three biological replicates. The results were analyzed by the 2^−∆∆Ct^ method (Ding et al., 2018; Liang et al., 2023).

### 2.8 Subcellular localization assay

The coding sequences of *AsbZIP14* and *AsbZIP41* were amplified using a Taq Plus DNA Polymerase kit (Tiangen, China) with primers listed in **Table S2** in *Escherichia coli* DH5α cells. The PCR program was set as follows: 5 min at 95 °C for initial denaturation, followed by 35 cycles with 95 °C for 10 s, 58 °C for 20 s, and 72 °C for 20 s. Then, the coding segments were respectively ligated into the pNC-Cam1304-SubC vectors containing green fluorescent protein (GFP) gene to generate pAsbZIP14-GFP and pAsbZIP41-GFP plasmids. *Agrobacterium tumefaciens* GV3101 strains harboring pAsbZIP14-GFP, pAsbZIP41-GFP, or pNC-Cam1304-SubC plasmids were separately grown to OD600 = 0.8 in LB medium at 200 rpm and 28°C and infiltrated into the onion epidermis by *Agrobacterium*-mediated transformation. The transformed onion epidermis was incubated in darkness at 27°C for 70 h. The GFP fluorescent signal of these onion epidermal cells was monitored using a Leica confocal fluorescence microscope (Wetzlar, Germany) after the nuclei were stained with 20 μg/mL 4´, 6-diamidino-2- phenylindole (DAPI) for 5 min and washed three times with the saline (Qu et al., 2020; Li et al., 2021a).

### 2.9 Yeast one-hybrid (Y1H) assay

The promoters of *AsPKS3* (1770 bp), *AsPKS4* (1770 bp), *AsPKS6* (1590 bp), *AsPKS8* (1380 bp), and *AsPKS9* (1335 bp) were amplified by PCR (**Table S2**) based on the other six AsPKS promoters containing abundant tandem TA or high GC content. The PCR products were separately inserted into bait vector pHIS2 with a Ready-to-Use Seamless Cloning Kit (Bhbio, China) to generate pHIS-pAsPKS3, pHIS-pAsPKS4, pHIS-pAsPKS6, pHIS-pAsPKS8, and pHIS-pAsPKS9 constructs. Meanwhile, the ORF sequence of *AsbZIP14* and *AsbZIP41* were respectively fused into the pGAD7 vectors to form the prey construct pGAD-AsbZIP14 and pGAD-AsbZIP41. Each of the bait vectors and the prey construct was transformed into yeast strain Y187 and the cells were cultivated on nutrient deficiency medium (SD/-His/-Leu/-Trp, SD-TLH) supplemented with different amounts of 3-amino-1,2,4-triazole (3-AT) at 30°C. The interaction between AsbZIP protein and the promoters of these *AsPKS* genes were scored after 3 days.

### 2.10 Dual-luciferase assays

The promoters of *AsPKS3*, *AsPKS4*, *AsPKS6*, *AsPKS8*, and *AsPKS9* were respectively cloned into pGreenII-0800-LUC plasmid and the ORF of *AsbZIP14* and *AsbZIP41* respectively ligated into pGreenII 62-SK to form the reporter vectors and effector vector. The resulting constructs and the empty pGreenII 62-SK vector (control) were individually transformed into *A. tumefaciens* GV3101 competent cells with pSoup-p19. *A. tumefaciens* cells were resuspended in infiltration medium (10 mM MES, 10 mM MgCl_2_ and 160 mM acetosyringone, pH 5.7) to a final OD600 = 0.8. The mixed bacterial cells of the effecter (1 mL) and reporter (3 mL) were infiltrated into *N. benthamiana* leaf tissues. The LUC and REN activities were detected using a Dual-Luciferase^®^ reporter assay system (Promega, USA) following the manufacturer’s instructions after the tobacco was infiltrated for 3 days (Li et al., 2021a). Experiments were performed using eight independent biological replicates and three technical replicates.

## 3 Results

### 3.1 Genome−scale identification of *bZIP* gene families in *A. sinensis* and eleven other species

**Figure 1:**
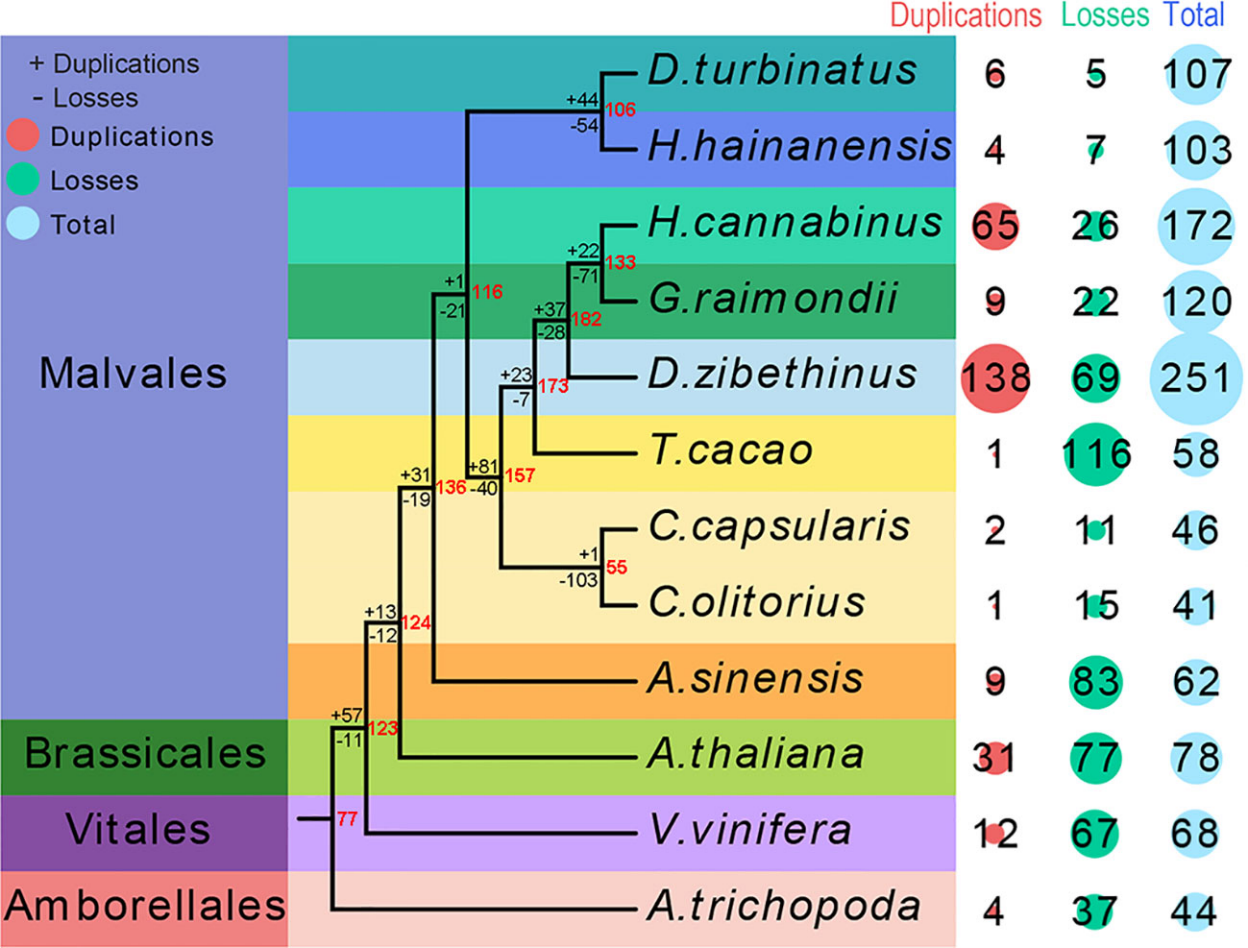
Quantitative distribution and duplication or loss analyses of *bZIP*s in nine Malvales plants and three model plants: *Arabidopsis thaliana*, *Vitis vinifera*, and *Amborella trichopoda*. The number of *bZIP* gene variations at different stages of plant evolution. The number of gene losses and duplications is indicated by “-” or “+” on each branch. The red number at node represents the number of bZIPs genes in ancestors species.

The seed proteins from the were non-redundant database of bZIP proteins from twelve species genomes used as a query to search against eleven species genomes to investigate the candidate *bZIP* genes in each species and uncover the evolutionary process of the *bZIP* gene family in Malvales. A total of 62 AsbZIP proteins were identified in *A. sinensis* genome and the results were verified by Pfam, CDD, and eggNOG databases (**Table S3**). The *bZIP* transcription factor gene family of three representative model plants and eight Malvales species was identified and verified using the same method. The number of *bZIP* genes in each genome were differed. A total of 251 *DzbZIP* genes were identified in *D. zibethinus*, followed by *H. hainanensis* (172), *G. raimondii* (120), *D. turbinatus* (107), *H. cannabinus* (103), *A. thaliana* (78), and *V. vinifera* (68). The three species without a recent whole genome duplication (WGD) event (*T. cacao*, *C. capsularis*, or *C. olitorius*) contained fewer *bZIP*s than *A. sinensis*. The species of *A. trichopoda* without WGD and whole genome triplication (WGT) only contained 44 *bZIPs* (**Figure 1**).

### 3.2 Evolution and differentiation of *bZIPs* in Malvales species

Subsequently, the phylogenies of these 12 species were reconstructed to reveal the evolutionary process of *bZIP* genes in Malvales using *A. trichopoda* as the outgroup species. The analysis of genes loss and duplication suggested that *bZIPs* gene family present rapid expansion in Malvales species experienced both WGD and WGT (**Figure 1**). Interestingly is that only 62 bZIPs genes in *A. sinensis* even this agarwood tree also undergone WGT and WGD. 77 ancestral genes were present in the lineage leading to the common ancestor of *A. trichopoda* and *V. vinifera*, and Malvales based on the duplication or losses of *bZIP* genes and the number of variations at different stages of evolution. Meanwhile, 57 ancestral genes were duplicated, and 11 ancestral genes were lost in the lineage leading to the common ancestor of *A. thaliana*, *V. vinifera*, and Malvales. Thirteen ancestral genes were duplicated, and 12 ancestral genes were lost for the branch of the common ancestor of *A. thaliana* and 9 Malvales species. The separation of *A. sinensis* from eight other Malvales species resulted in the duplication of 31 ancestral genes and the loss of 19 ancestral genes. This result suggests that the duplication or losses of *bZIP* gene families in each Malvales species are not uniform since the divergence. Nine out of sixty-two *bZIP* genes in *A. sinensis* originated from duplication, while 83 members were lost in species evolution or differentiation. In summary, more *bZIP* genes were lost than duplicated in *A. trichopoda*, *A. thaliana*, *V. vinifera*, and Malvales species except in *D. zibethinus* and *H. cannabinus*. This result implied that the *bZIP* gene families of *D. zibethinus* and *H. cannabinus* were significantly larger than that of other species (**Figure 1**).

**Figure 2:**
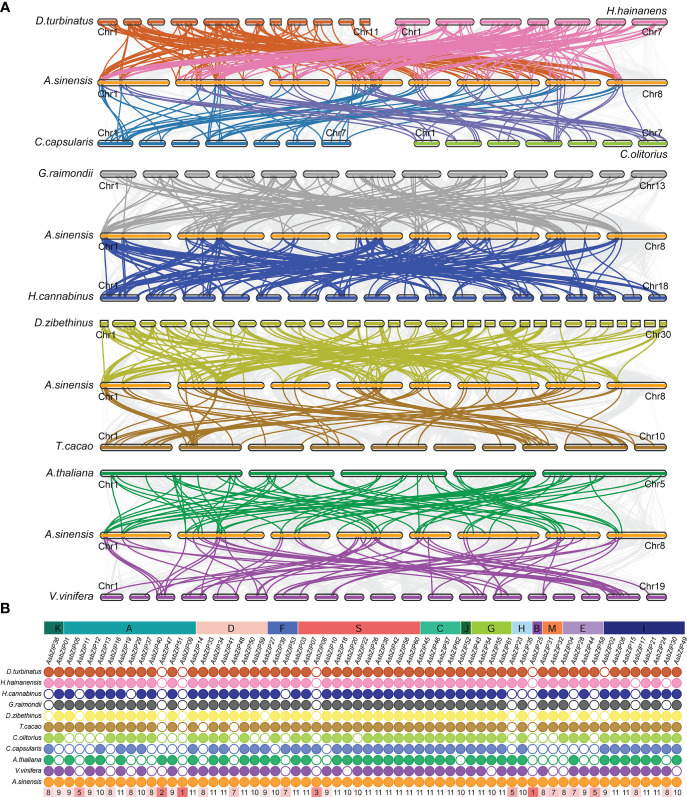
Synteny relationship of *AsbZIP*s with *bZIP*s from eight other Malvales plants, *A. thaliana*, and *V.vinifera*. **(A)** Synteny analyses of AsbZIPs and *bZIPs* from other species. **(B)** The statistics of collinear relationship. Gray lines in the background indicated the syntenic blocks within *A. sinensis* and other plant genomes. The highlighted lines indicate the *AsbZIP*s with their collinearity gene in different species. The number in red rectangle show the number of species holding the orthologous gene of this *AsbZIP*.

The comparative synteny and collinearity maps of *bZIP* genes were constructed using eleven species (excluding *A. trichopoda* because it lacks a chromosome-level genome) to identify the orthologous *bZIPs* genes between *A. sinensis* and other species. There were 136, 105, 124, 114, 155, 68, 63, 59, 42, and 49 orthologous pairs between *A. sinensis* and the other ten species (*D. zibethinus, H. hainanensis, G. raimondii, D. turbinatus, H. cannabinus, A. thaliana, T. cacao, V. vinifera, C. olitorius, and C. capsularis*, respectively) (**Figure 2A**). Only *AsbZIP09* and *AsbZIP25* had no orthologous pairs with the other 11 species; this indicated that they might be unique to *A. sinensis* (**Figure 2B**). The *bZIP*s were more conserved in these species.

**Figure 3:**
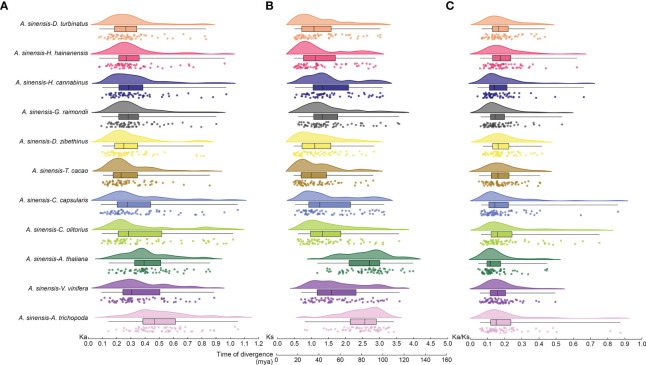
Ka, Ks, and Ka/Ks analysis, and divergence time of *bZIP* syntenic gene pairs in *A. sinensis* and other species. **(A)** Nonsynonymous (Ka) values. **(B)** Synonymous (Ks) values and divergence time. **(C)** The Ka/Ks ratio. Different species were plotted using different colors, while the same plant was drawn with the same color.

Nonsynonymous (Ka) and synonymous (Ks) values for *bZIP* gene pairs between *A. sinensis* and other eleven selected species were calculated to detect the driving force of the *bZIP* gene family evolutionary process (**Figures 3A, B**). The Ka/Ks value ranged from 0.04–1.18 and all of the Ka/Ks ratios were < 1 except for one pair (*AsbZIP45* and *AsbZIP46*) (**Figure 3C**). All Ka/Ks values of *bZIP* gene pairs between *A. sinensis* and eleven other species were significantly below 1. This indicated that the *bZIP* genes were subjected to negative selection and high conservation in plant evolution. The Ka/Ks value of the collinearity pairs of *AsbZIP45* and *AsbZIP46* was above 1 within the *A. sinensis* genome, whereas the Ka/Ks values of 15 other paralogous *AsbZIP* gene pairs were below 1. This confirmed that the *AsbZIP* gene family primarily underwent negative selection in the subsequent evolution of *A. sinensis*.

The locations of their means, variances, and peaks were statistically determined after using the normal distribution function to fit the Ks distribution. The Ks peaks from the *bZIP* gene families of different species were in different positions, while some species had similar Ks peaks and shared a similar evolutionary rate (**Figure 3B**). The Ks peak of *bZIP*s in *A. trichopoda*, *A. thaliana*, and *A. sinensis* was 3.08, 2.97, and 1.10, respectively. The Ks peak of *bZIPs* from *H. hainanensis* was similar to that of *H. cannabinus* and *G. raimondii*, while the *bZIP* Ks peak of *D. turbinatus* was similar to that of *D. zibethinus* and *T. cacao*. Besides, the Ks peak of *bZIPs* in *V. vinifera* resembled that of *C. olitorius* and *C. capsularis*. This might be caused by the retention of ancient WGD events and the mission of the recent WGD events in these three species.

The Ks values were further used to track the divergence time of these gene pairs for the 11 species to deduce the divergence time between *bZIP* genes of different species. The divergence time of *bZIP* genes in *A. sinensis* extended from 36.00–102.06 million years ago (Mya) except a duplication event of *AsbZIP45* and *AsbZIP46* occurred at 0.25 Mya, which was dividing during the recent WGD. The divergence time of *bZIP* genes in *A. sinensis* was approximately 26.69–122.32 (Mya) compared with eight other Malvales species. Meanwhile, the divergence time of *bZIP*s in *A. sinensis* occurred at ~ 48.18–134.95 Mya, ~ 33.53–122.66 Mya, and ~ 36.89–117.46 Mya, respectively compared with *A. thaliana*, *V. vinifera*, and *A. trichopoda* (**Figure 3B**). These results showed that the divergence times of *bZIP* genes in *A. sinensis* and other Malvales are closer compared with other species. These results suggested that the divergence of the common ancestor *bZIP* genes in these species occurred near the core eudicot-common hexaploidy (ECH) event at ~ 115–130 million years ago (Mya) and the *bZIP* genes were the ancient and conserved transcription factors in angiosperm evolution.

### 3.3 Evaluation of chromosomal distribution and synteny analysis of *AsbZIPs*

**Figure 4:**
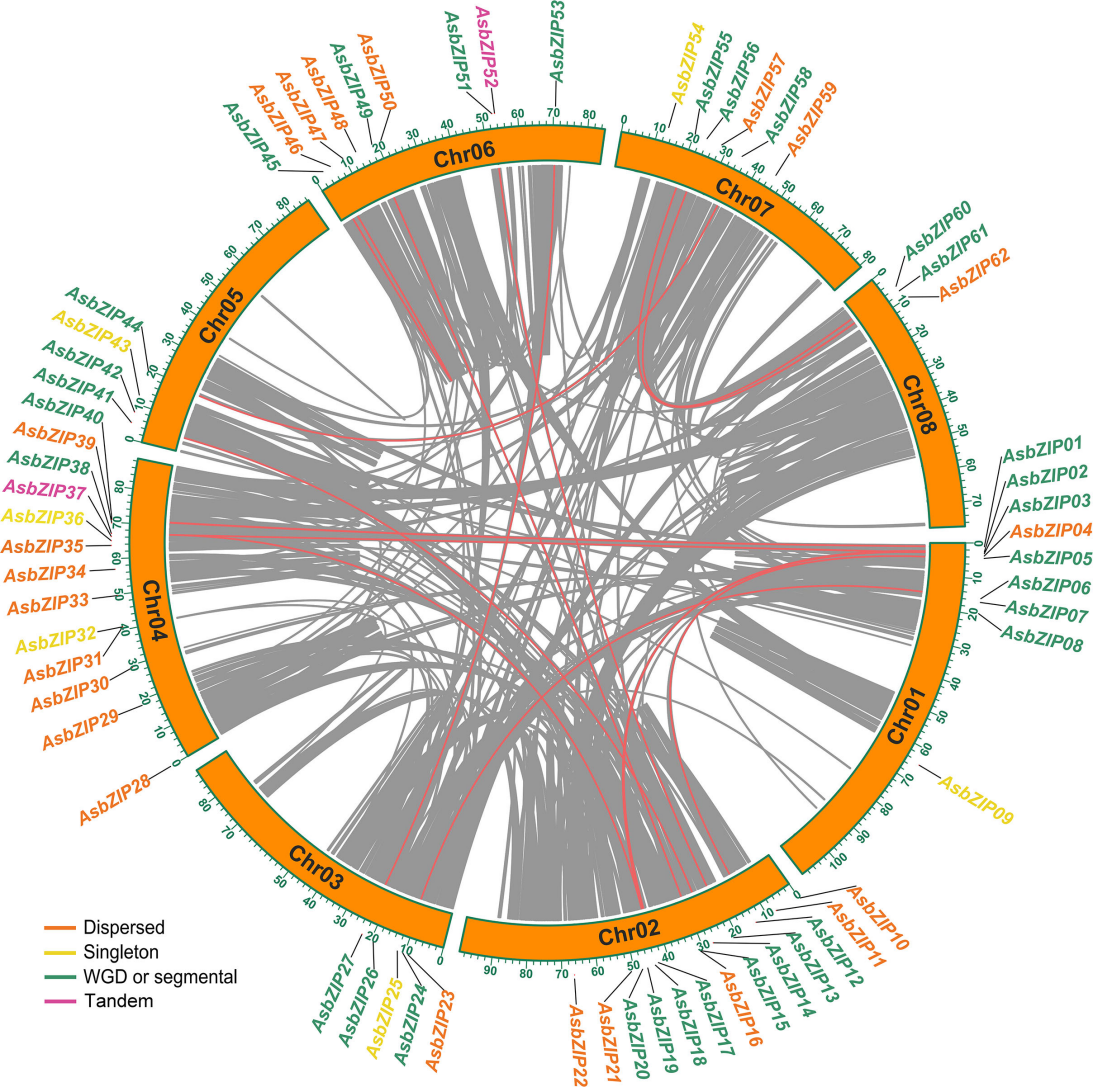
Chromosomal distribution and synteny analysis of *AsbZIP* genes. Gray lines represented syntenic blocks in the agarwood genome and red lines indicate syntenic *AsbZIP* gene pairs. Five gene duplication modes of *AsbZIP* gene names were highlighted with different colors in the external circles.

A joint analysis of *bZIP* genes in the *A. sinensis* genome was performed using Blastp and MCScanX to further explore the expansion mechanism of the *bZIP* gene family. The 62 *AsbZIP* genes were unevenly distributed on eight chromosomes of *A. sinensis*: Chr 8 contained only three *bZIP* genes, while Chr 2 and Chr 4 harbored 13 *AsbZIPs* each. In addition, Chr 1 and Chr 6 possessed 9 *AsbZIP* genes each, while Chr 7, Chr 3, and Chr 5 contained 6, 5, and 4 *AsbZIP* genes, respectively (**Figure 4**).

In order to identify relationships among the *AsbZIP* genes and define the potential gene duplication events, paralogous information was examined and 16 paralogous *AsbZIP* gene pairs (27 *AsbZIP* gene and one other gene) located on different chromosomes were found in the *A. sinensis* genome. One paralogous gene (evm.model.Scaffold68.87) has no *bZIP* conserved domain and can be removed from *AsbZIP* gene family. This is an example of the fact that the structure of a gene may be changed during the duplication events. The percentage of paralogous *AsbZIP* genes was 43.5% (27 paralogous genes in 62 *AsbZIPs*). This indicated that duplications were probably conducive to the expansion of the *AsbZIP* family. Therefore, five gene duplication modes (WGD or segmental, tandem, proximal, singleton, and dispersed duplications) of 62 *AsbZIP* genes were examined to understand different gene duplication contributions to the expansion of *AsbZIP* gene family. The percentage of WGD or segmental *AsbZIPs* was 51.6% (32), followed by dispersed duplication (35.5%, 22), singleton (6, 9.7%), and tandem (2, 3.2%); proximal duplication was not detected (**Table S4**). Briefly, the origin of the *AsbZIP* gene family occurred by the WGD or dispersed duplication in *A. sinensis*.

### 3.4 Phylogenetic analysis and classification of *AsbZIPs*

**Figure 5:**
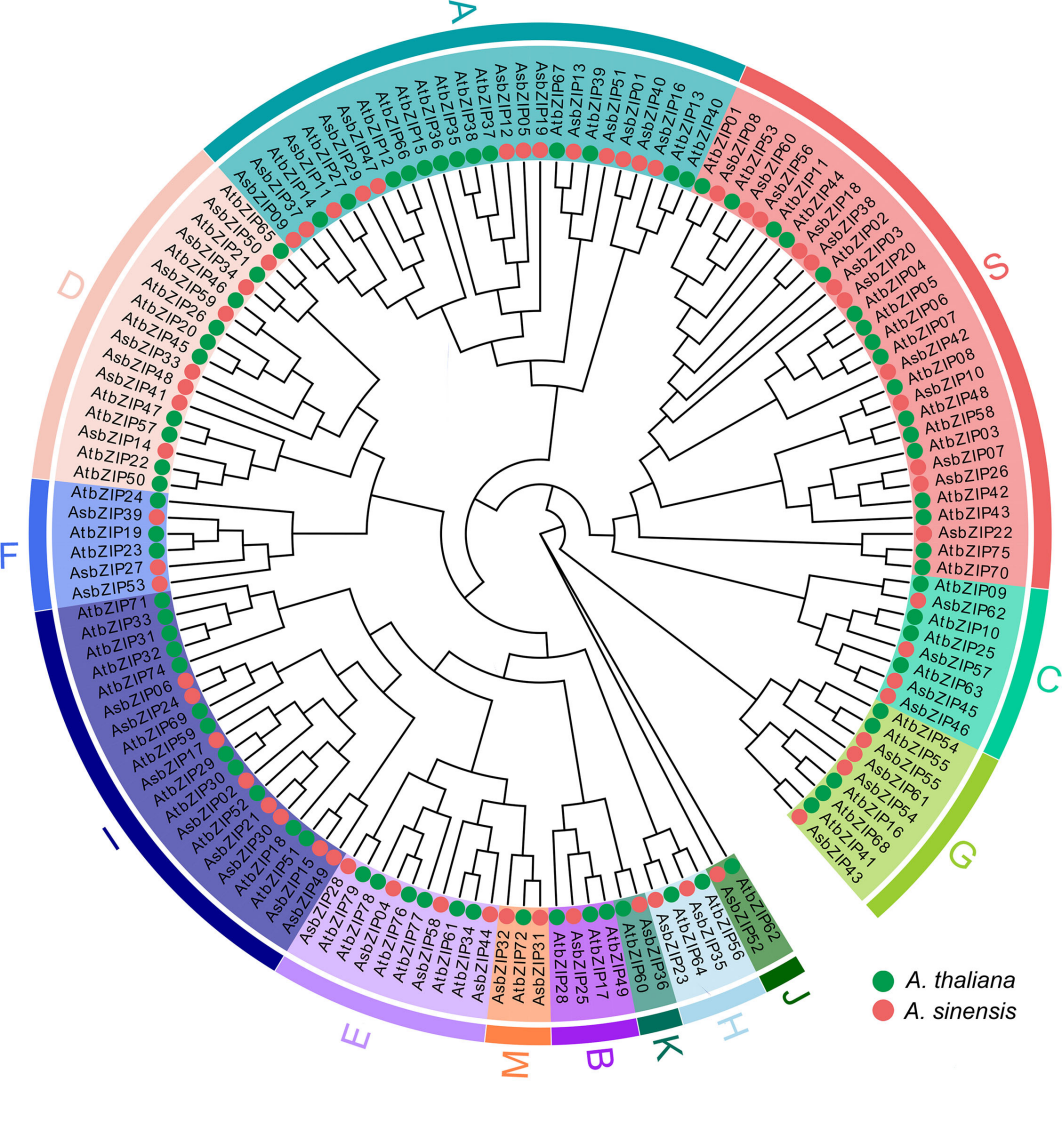
Phylogenetic tree of the *bZIP* gene family in *A. sinensis* and *A. thaliana*. Iqtree was used for model selection, followed by the selection of the best model (VT+F+R7) to generate the phylogenetic tree. The classification into 13 groups (A-K, M, and S) was marked by different colors.

**Figure 6:**
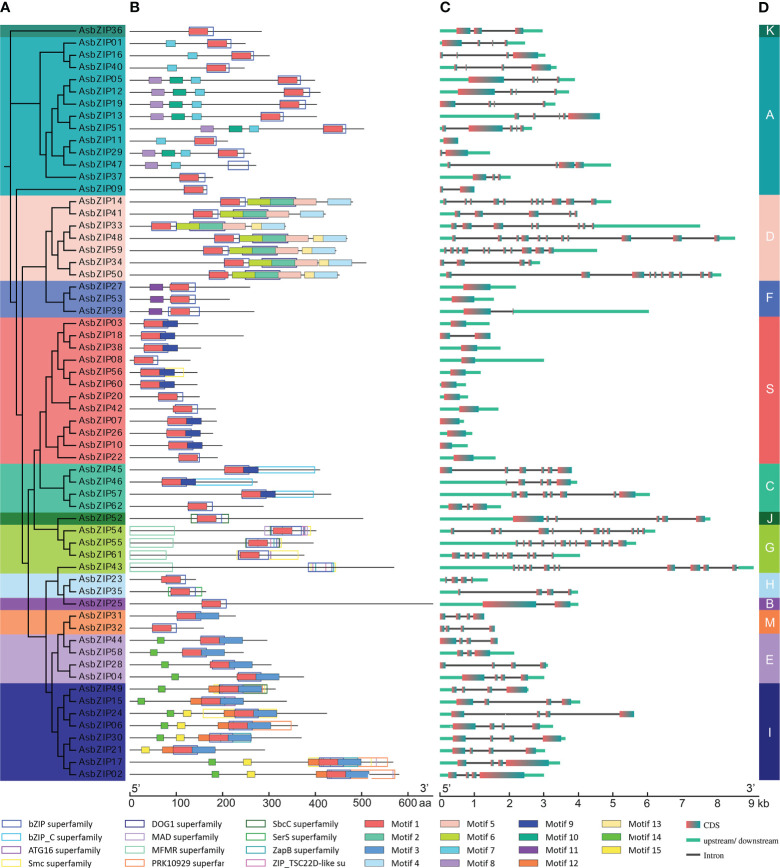
Phylogenetic relationship, conserved domains, motif pattern, and exon–intron structure of AsbZIP proteins and genes. **(A)** Phylogenetic analysis of AsbZIP proteins. **(B)** Functional domain and motif compositions of AsbZIP proteins. Fifteen motifs were indicated with rectangles using different fill colors. Twelve conserved domains were shown in rectangles with different outline colors, and their detailed information is provided in **Table S6** and **Figure S1**. **(C)** Exon-intron structures of *AsbZIP* genes. **(D)** Organization of the AsbZIP family. Thirteen subfamilies are marked by different colors.

The phylogenetic tree was constructed based on the protein sequences of 62 AsbZIPs and 78 AtbZIPs to explore the phylogenetic relationship and subfamily classification of AsbZIPs proteins (**Figure 5**). Sixty-two AsbZIPs were divided into 13 subfamilies (A-K, M, and S); this is the same as bZIPs in *A. thaliana* based on conserved domains and motifs (**Figures 5**, **6A**). A and S are the largest subfamilies with 13 and 12 members, respectively, followed by the I subfamily (8), D subfamily (7), E subfamily (5), C subfamily (4), G subfamily (4), F subfamily (3), and H subfamily (2); meanwhile, the B, J, K, and M subfamilies contained only one member each.

The lengths of AsbZIPs ranged from 130 AA (AsbZIP08) to 663 AA (AsbZIP25). The predicted molecular weight (MW) of the 62 AsbZIPs was between 15.26 kDa (AsbZIP08) to 72.34 kDa (AsbZIP25). All AsbZIP proteins were predicted to be hydrophilic and the isoelectric point (pI) was predicted to be between 4.77 (AsbZIP36) to 10.69 (AsbZIP37) (**Table S5**). Almost all AsbZIP proteins were predicted to localize to the nucleus, except for AsbZIP25 (endoplasmic reticulum) and AsbZIP32 (chloroplast) (**Table S5**).

Protein sequences of 62 AsbZIPs were analyzed using CDD and MEME for the detection of conserved domains and putative motifs to further confirm and understand the composition and gene structure of AsbZIPs (**Figure 6B**, **Figure S1** and **Table S6**). All AsbZIP proteins almost contain motif 1 which has a specific N-X7-R/K motif that is regarded as a basic DNA-binding region of bZIP proteins, and an adjacent so-called leucine zipper (**Figure 6B**). The leucine zipper is a domain region that has characteristic heptad repeats of leucine (L) or related hydrophobic amino acids and is general associated with enabling dimerization (Vinson et al., 1989). AsbZIP43 and AsbZIP47 have no motif 1; however, it is hard to eliminate them from the bZIP family since each of them contains the same conserved bZIP superfamily domain.

Exon-intron organization was sequentially investigated to comprehensively understand the similarity and diversity of *AsbZIP* genes. The number of introns in the *AsbZIPs* ranged from 0 to 11 out of 13 groups of intron/exon structures (**Figure 6C**). The D and G subfamilies are the most intron-rich genes compared with the other subfamilies and contained 7–14 introns, except for *AsbZIP34* and *AsbZIP41* since they had incomplete gene structures. *AsbZIP14* and *AsbZIP4*1 of subfamily D had the closest relationship (**Figure 6A**), although they contained 10 and 4 introns, respectively (**Figure 6C**). This suggested that their expression and the regulatory mechanism were different. However, no intron was found in the S and F subfamilies except for *AsbZIP18* and *AsbZIP39* which contained one intron each. Furthermore, one member in the B subfamily (*AsbZIP25*) contained only one intron. Meanwhile, almost all of the eight other subfamilies presented 3-4 introns. These results indicated that *AsbZIPs* classified into the same subfamily shared a highly similar composition and position of introns/exons, but different subfamilies had large variations.

The functional diversity of the AsbZIP family might be initially predicted based on the experimentally tested function of *Arabidopsis* bZIP subfamilies and the diverse conserved domains of each subfamily. The plausible proposition is that the proteins categorized within the different groups tended to perform different functions according to phylogenetic tree analysis combined with structural diversity, motif prediction, and domain map of AsbZIPs (**Figures 5**, **6**) (Hu et al., 2016; Unel et al., 2019; Rong et al., 2020).

### 3.5 Analysis of *cis* elements in the *AsbZIP* promoters

The *cis* acting elements in the promoter of *AsbZIP* genes were analyzed using the Plantcare database. A total of 51 *cis* acting elements related to light response (30), plant development (7), hormone response (8), and related to environmental stress (6) were further analyzed (**Figure S2**). All of the *AsbZIPs* promoters contained light response element, which is the most abundant and kinds of element. Multiple-stress responsive elements are common in the promoters of all *AsbZIPs*, except for *AsbZIP10* and *AsbZIP44*. Among them, 27, 54, 25, 27, and 18 *AsbZIPs* contained wound-responsive elements (WUN-motif), anaerobic induction elements, or enhancer-like element involved in anoxic specific inducibility (ARE and GC-motif), low-temperature responsive elements (LTRs), drought inducibility elements (MBS), and defense and stress-responsive elements (TC-rich repeats), respectively. We also found multiple-hormone responsive elements in the *AsbZIP* family, and these plant hormone response-related acting *cis* elements were distributed in almost all members except *AsbZIP11* and *AsbZIP49*. Meanwhile, 26, 50, 39, 21, and 38 *AsbZIPs* contained salicylic acid (SA) *cis* elements (TCA-element), abscisic acid responsiveness *cis* elements (ABRE), methyl jasmonate (MeJA) *cis* elements (TGACG-motif and CGTCA-motif), auxin *cis* elements (TGA-element and AuxRR-core), and gibberellin *cis* elements (GARE-motif, TATC-box, and P-box), respectively. In addition, 46 members of the *AsbZIP* family contained plant growth and development-related *cis* elements. Namely, 5, 4, 10, 26, 15, and 24 *AsbZIPs* contained *cis* acting elements involved in cell cycle regulation (MSA-like), *cis* elements responding to the differentiation of palisade mesophyll cells (HD-Zip 1), circadian control *cis* elements (circadian), meristem expression *cis* elements (CAT-box), endosperm expression *cis* elements (GCN4_motif), and *cis* acting elements involved in zein metabolism regulation (O2-site), The promoters of *AsbZIP07* harbored a *cis* element with an AACA_motif involved in the endosperm-specific negative expression. Together, these results indicated that *AsbZIPs* may respond to many different kinds of light, growth and development, and stress and hormone signaling. These results suggested that *AsbZIP* family genes might have various functions and each subfamily might play different roles in plant developmental events, and biotic and abiotic responses.

### 3.6 Analysis of genes nearby *AsbZIPs*

Five upstream genes and five downstream genes around 62 *AsbZIP*s were further analyzed to investigate the potential effect of nearby genes on *AsbZIP*s. Sixty-one pseudo gene clusters were constructed ranging between 65.59–900.93 kb according to *AsbZIP31* and *AsbZIP32* tandem genes on the same chromosome (**Figures S3**, **S4**). A total of 671 genes (62 *AsbZIPs* and 609 nearby genes) were enriched into 21 COG categories (**Figure S3**). Transcription (103, 15.35%) was enriched (except in the largest unknown functional categories: 295, 43.96%), followed by posttranslational modification, protein turnover, and chaperones (37, 5.51%), signal transduction mechanisms (26, 3.87%), translation, ribosomal structure, and biogenesis (25, 3.73%), energy production and modification (21, 3.13%), and carbohydrate transport and metabolism (19, 2.83%) (**Figure S3**). Different from WGD or segmental had the greatest contributions to the expansion of *AsbZIP* gene family, more dispersed duplications (269, 40.09%) were discovered in nearby genes of *AsbZIPs*, followed by WGD or segmental (181, 26.97%), singleton (175, 26.08%), tandem (36, 5.37%) and proximal duplications (10, 1.49%) (**Figure S4**). These results suggested that nearly all genes distributed around *AsbZIP*s were none functional gene directly involved in secondary metabolism while they might provide regulatory function via protein interaction with DNA elements.

### 3.7 Expression profiles of *AsbZIP* genes during agarwood formation

**Figure 7:**
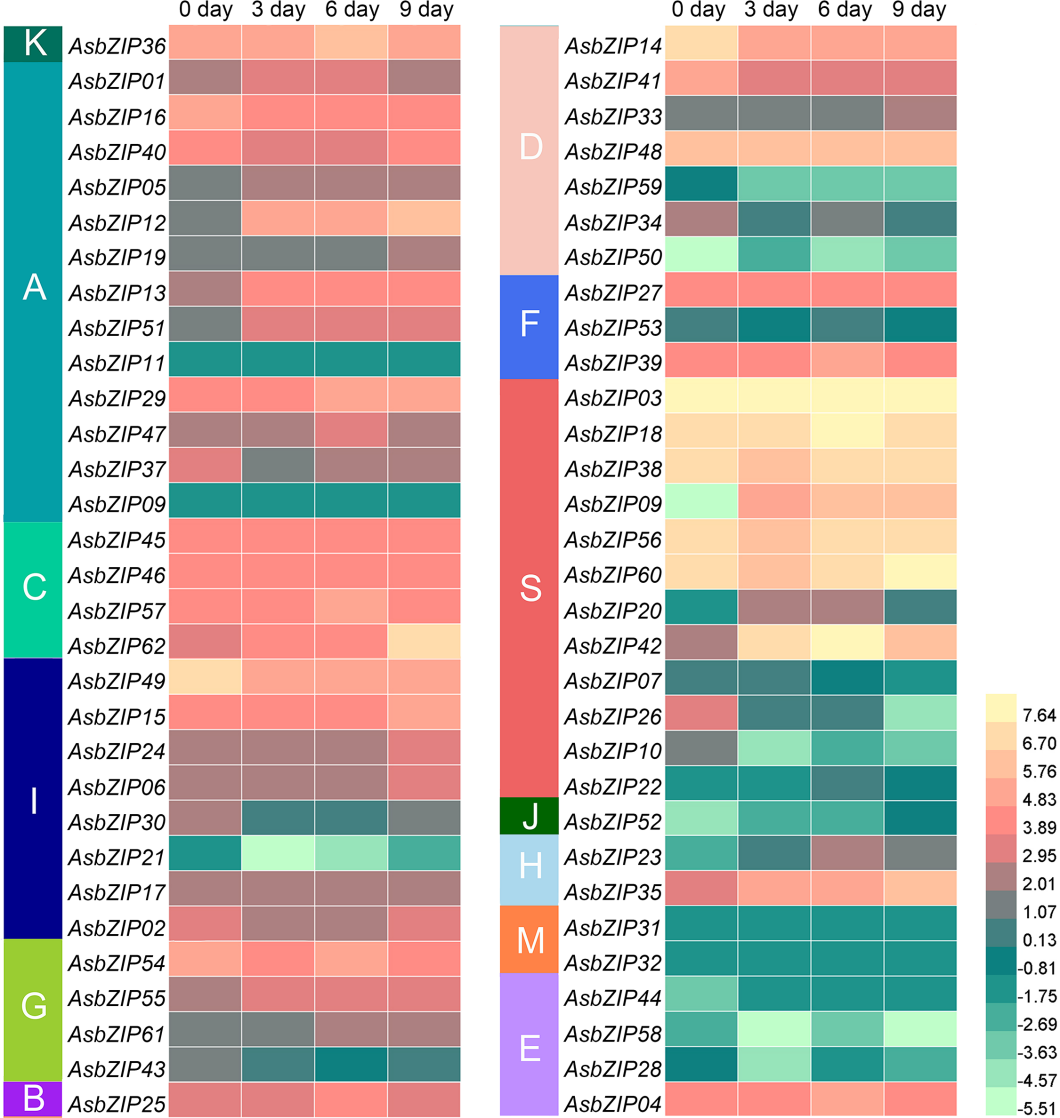
Expression profiles of *AsbZIP* genes in *A. sinensis* stems treated with agarwood-inducer. The 0 day, 3 day, 6 day, and 9 day tags indicated the time-points after the stems of *A. sinensis* were treated with the agarwood inducer. The RPKM (reads per kilobase of exon model per million mapped reads) were transformed to log2.

The expression levels of 62 *AsbZIP* genes were analyzed to further distinguish candidate *AsbZIP* genes related to PEC biosynthesis (**Figure 7**). The expression profiles of *AsbZIP* genes are significantly different during agarwood formation. Five *AsbZIPs* had no transcript abundance (*AsbZIP09, 11, 31, 32*, and *44*), while the other 57 *AsbZIPs* were expressed in this process (32 were upregulated, while 25 were downregulated). Transcriptomic analysis showed that there *12 AsbZIP* genes (*03*, *08*, *14*, *18*, *35*, *38*, *41*, *42*, *49*, *56*, *60*, and *62*) were expressed at high levels and *AsbZIP03* was significantly expressed. Both candidates of subgroup M (*AsbZIP31* and *32*) and all members of subgroup E had low expression except *AsbZIP04*. The expression of subfamily S *AsbZIP*s was starkly polarized on account of seven genes out of twelve *AsbZIPs* showing high expression levels compared with all *AsbZIPs*; however, the last five genes were expressed at relatively low levels. Subfamily D contained seven members, with four genes showing low expression during agarwood formation, while *AsbZIP48* was consistently expressed. Meanwhile, *AsbZIP14* and *AsbZIP41* had high expression levels, this suggests that they might be involved in regulating type III *PKS* expression.

**Figure 8:**
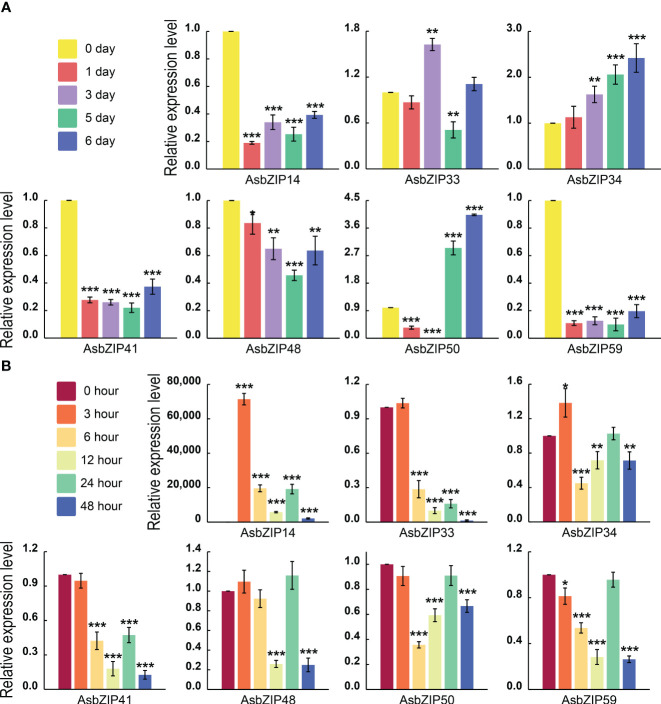
Relative expression of *AsbZIP* genes of subfamily D in *A. sinensis* stems after treatment with agarwood inducer and ethylene (ET). **(A)** Relative expression of *AsbZIP* genes of subfamily D in the *A. sinensis* stem after treatment with agarwood inducer at day 0, 1, 3, 5, and 6. **(B)** Relative expression of *AsbZIP* genes of subfamily D in *A. sinensis* stems after treatment with ET at 0, 3, 6, 12, 24, and 48 **(h)** qRT-PCR data from three independent biological replicates were shown using standard errors (SE) and *AsHistone* was used as the internal control. Asterisks represent significant differences (*p < 0.05; **p < 0.01; ***p < 0.001).

The expression levels of all *AsbZIP* genes from subfamily D were detected by qRT-PCR following ethylene (ET) and agarwood-inducer treatment to distinguish PEC biosynthesis-related *AsbZIPs* genes in subfamily D (**Figure 8**). Ethylene stimulus increased *AsbZIP1* expression by 71,427.18-fold compared with the control at 3 h, followed by a decrease after 48 h, with a small peak at 24 h. The expression of *AsbZIP41* and *AsbZIP33* was also significantly induced by ET (**Figure 8A**). In comparison, the expression of *AsbZIP14*, *AsbZIP41*, and *AsbZIP59* was significantly down-regulated under the agarwood-inducer treatment. The agarwood-inducer treatment caused a decrease in *AsbZIP50* expression and reached the lowest level at six hours. This was followed by a considerable increase to its highest level at 48 hours (**Figure 8B**).

### 3.8 The regulatory mechanism of *AsbZIP14* and *AsbZIP41* interacting with type III *AsPKS*

**Figure 9:**
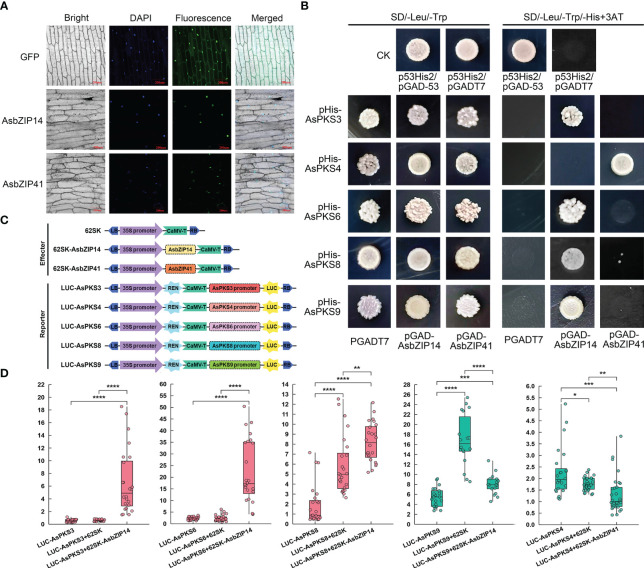
Promoter activation of *AsPKS3*, *AsPKS4*, *AsPKS6*, *AsPKS8*, and *AsPKS9* by *AsbZIP14* and *AsbZIP41*. **(A)** Nuclear localization of *AsbZIP14* and *AsbZIP41* in onion epidermal cells. **(B)** Yeast one-hybrid analysis of *AsbZIP14* and *AsbZIP41* proteins binding to the promoters of five *AsPKSs*. Assays were repeated three times. **(C)** Schematic diagrams of the effecter and reporter plasmids used in Dual-LUC assays. REN, *Renilla* luciferase internal reference gene. LUC, firefly luciferase reporter gene. **(D)** Dual-luciferase (Dual-LUC) assays showed that *AsbZIP14* or *AsbZIP41* regulated the transcriptional activation of the promoters of five *AsPKS*s. The suppression and promotion effect were colored green and red, respectively. The activities of LUC and REN were determined 3 days after infiltration. The data were indicated by the ratio of LUC to REN and the units of y-asix are 1×e^-2^.. Data represent the mean ± SE of eight biological replicates and three technical replicates. Significant differences were tested by analysis of variance (ANOVA: *p < 0.05; **p < 0.01; ***p < 0.001, ****p < 0.0001).

The subcellular location of *AsbZIP14* and *AsbZIP41* was analyzed to investigate their roles in regulating chromone biosynthesis by activating *AsPKS* expression. The GFP fluorescence produced by *pAsbZIP14*-GFP or *pAsbZIP41*-GFP was preponderantly located in the nucleus (**Figure 9A**). This demonstrated that *AsbZIP14* and *AsbZIP41* perform their regulatory function as TFs in the nucleus. Next, Yeast one-hybrid (Y1H) assay was used to verify the ability of *AsbZIP14* and AsbZIP41 interacted with the promoter of *AsPKS3*, *AsPKS4*, *AsPKS6*, *AsPKS8* and *AsPKS9.* The results of Y1H assays confirmed that *AsbZIP14* physically interacted with the promoter of *AsPKS3*, *AsPKS6*, *AsPKS8*, and *AsPKS9* and might regulate their transcription; however, it did not interact with the AsPKS4 promoter (**Figure 9B**). Meanwhile, *AsbZIP41* presented a completely different function (**Figure 9B**). The dual-luciferase reporter gene assay (Dual-LUC) was conducted to better understand the *AsbZIP14* and *AsbZIP41* mode of action on *AsPKS* genes (**Figure 9C**). It further verified the binding of *AsbZIP14* with the promoter regions of *AsPKS3*, *AsPKS6*, *AsPKS8*, and *AsPKS9*, respectively (**Figure 9D**). *AsbZIP14* exhibited an 11.0-fold increase in the activity of the *AsPKS3* promoter and a 9.7-fold increase in the activity of the *AsPKS6* promoter. Activation of the *AsPKS8* promoter was also suppressed by *AsbZIP14* with a 1.6-fold increase. Meanwhile, the expression of *AsPKS9* resulted in a more than 1.5 folds decrease in the luciferase activity controlled by *AsbZIP14*. The level of the luciferase activity controlled by *AsbZIP41* and *AsPKS4* promoters was suppressed more than 1.6 folds suggested that *AsbZIP41* could transcriptionally downregulate *AsPKS6*. (**Figure 9D**). *AsbZIP14* could activate the expression of *AsPKS3*, *AsPKS6* and *AsPKS8* whereas that could inhibit the expression of *AsPKS9* based on Dual-LUC assays and Y1H assays. Besides, the *AsPKS4* promoter was also mediated by *AsbZIP41*.

## 4 Discussion

### 4.1 Molecular evolution of *bZIPs* in Malvales

There is an increasingly systematic and comprehensive identification of the *bZIP* gene family of different plants with high-quality plant genomes in recent years. Nevertheless, the exhaustive analyses and functional characterization of *bZIP* families in Malvales plants are unavailable. This work provided a detailed and systemic analysis of *AsbZIP* genes in *A. sinensis* and estimated *bZIP* candidates of eight other Malvales plants (cocoa, *C. capsularis*, *C. olitorius*, cotton, durian, *D. turbinatus*, *H. hainanensis*, and kenaf) with *A. thaliana*, grape, and *A. trichopoda* used as the references.

The bZIPs are ancient and conserved transcription factors in plant evolution; however, their numbers in each plant are different and diverse (Weirauch and Hughes, 2011; Dröge-Laser et al., 2018). In this study, 1150 *bZIP* candidate genes were identified in eight Malvales species, a model plant and two species at the key nodes of plant evolution. This included, 62, 78, 120, 251, 58, 46, 41, 107, 103, 172, 68, and 44 *bZIP* genes in agarwood tree, *A. thaliana*, cotton, durian, cocoa, *C. capsularis*, *C. olitorius*, *D. turbinatus*, *H. hainanensis*, kenaf, grape, and *A. trichopoda*, respectively. Polyploidization might always contribute to the gene functions differentiation and number change (Song et al., 2018; Meng et al., 2021; Hou et al., 2022). Duplications and gene losses in this process are the important driving forces for shaping *bZIP* families The differences in *bZIP* genes in numerous species might result from gene duplication or losses to various degrees (**Figure 1**). The ancestry of all angiosperms suffered from an ancestral whole genome triplication (WGT), followed by an additional recent WGD event that affected agarwood tree, cotton, durian, *D. turbinatus, H. hainanensis*, and *H. cannabinus* (Vanneste et al., 2014). In contrast, no additional recent polyploidization event was found in cocoa, *C. capsularis*, *C. olitorius*, grape, and *A. trichopoda* (**Figure 3B**) (Argout et al., 2011; Guan et al., 2014; Zheng et al., 2015; Canaguier et al., 2017; Lian et al., 2018; Wang et al., 2019; Meng et al., 2021; Zhang et al., 2021; Wang et al., 2022b). Interestingly, the number of *bZIP* genes in *A. sinensis* was similar to that of cocoa and grape which only experienced the angiosperms-common hexaploidy, but was noticeably less than Malvales experienced additional WGD. The distinct result might be caused by *A. sinensis* is the relative ancient species in Malvales. Based on these results, we speculate that more *AsbZIP* candidates were lost during the evolution of *A. sinensis* compared with other analyzed Malvales species.

The divergence time analysis agreed that the ancient ancestor genes of *bZIPs* in these species occurred during the core eudicot-common hexaploidy (ECH); therefore, *bZIPs* were an evolutionarily ancient gene family for angiosperms (**Figure 3B**). In addition, collinear analysis of agarwood tree, *Arabidopsis*, grape, cotton, cocoa, *C. capsularis, C. olitorius*, durian, *D. turbinatus*, *H. hainanensis*, and kenaf showed the evolutionary conservation of the *bZIP* family between Malvales and non-Malvales plants (**Figure 2**). These results were consistent with previous analyses of *bZIPs* in green plants. The observed purifying selection of the *AsbZIP* gene family is supported by the fact that most Ka/Ks values of paralogous *AsbZIP* gene pairs were below 1 (**Figure 3C**). Moreover, analysis of *AsbZIP* gene duplication modes showed that *bZIP* gene expansion was mainly derived from WGD or segmental duplication (**Figure 4**).

### 4.2 The potential functional analysis of bZIPs in *A. sinensis*

The basic region of bZIP protein consists of a DNA-binding region that is an invariant N-X_7_-R/K motif with asparagine (N) and basic (R/K) residues with exact spacing(Dröge-Laser et al., 2018). However, sites that contained variations in the N and R/K residues were also considered *bZIP* genes. For instance, the bZIP 76-bZIP79 in subfamily E of *A. thaliana* harbors a deletion or even lacks the N residue (Corrêa et al., 2008; Dröge-Laser et al., 2018). In this study, the six AsbZIPs (AsbZIP04, AsbZIP09, AsbZIP28, AsbZIP32, AsbZIP43, and AsbZIP51) identified with this method contained the conservative bZIP domain and variations in the N or R/K residues (**Figure S1**). The close relationship between these six AsbZIPs with the typical bZIPs in *A. sinensis* or *A. thaliana* suggests that more details should be considered to identify the *bZIP* gene family in other plants.

Enormous functional analysis of bZIPs was generated in the model plant, *A. thaliana*. Most of the results determined a prototypic model to define the subfamily classification and predict the function of *bZIP* genes in each subfamily (Zheng et al., 2015; Duan et al., 2022; Zhang et al., 2022a). Sixty-two AsbZIPs from the agarwood tree genome were sorted into 13 groups similar to bZIP in *A. thaliana* according to the phylogenetic tree and the conserved motif analysis compared with previously identified and classified gene families (**Figure 6**) (Dröge-Laser et al., 2018). The group-specific functional and regulatory properties of AsbZIPs were predicated according to the exhaustive summary of bZIPs in *A. thaliana* and multitudinous angiosperm. Specifically, most bZIP members in subfamily A directly bind to abscisic acid responsive *cis*-elements (ABRE) and are involved in the abscisic acid pathway to counteract water deficit, floral transition control, and seed development (Banerjee and Roychoudhury, 2017; Shu et al., 2018; Collin et al., 2020). Group B and K members were involved in regulating stress response in the endoplasmic reticulum (Kim et al., 2018; Ruberti et al., 2018). The bZIP members of subgroup C preferentially heterodimerize with group S1 members and form the C/S1-bZIP network that always functions as signaling hub genes to manage plant energy; this leads to species survival from environmental stress and metabolic adaptation (Weltmeier et al., 2009; Pedrotti et al., 2018; Li et al., 2020). Moreover, group S always comprises many bZIP members to benefit plant survival following various abiotic and biotic stresses, including salt stress, cold stress, bicarbonate alkaline stress, extended darkness, and pathogen defense responses (Fu and Dong, 2013; Weiste et al., 2017; Wu et al., 2018; Shine et al., 2019; Wang et al., 2022a). Subfamily D bZIP members (also known as TGA factors) control various important signaling molecules of the phytohormone transduction pathway. They physically interact with genes that are crucial participants of plant innate pathogen responses and are regulators in systemic acquired resistance; this is a broad-spectrum immunity triggered by a prior local pathogen infection at the whole plant level (Dröge-Laser et al., 2018). For example, AtTGA3 mediates the interaction between SA and cytokinin and prevents pathogen damage (Choi et al., 2010); TGA2, TGA5, and TGA6 evoke necrotrophic pathogen defense responses activated by jasmonic acid (JA) and ET in *A. thaliana* (Zander et al., 2010); while TGA1 and TGA4 induce defense mechanisms against bacterial pathogens in *A. thaliana* (Wang and Fobert, 2013). AcTGA01, AcTGA06, and AcTGA07 are responsive to hormones at different levels and increase their resistance to kiwifruit canker caused by pathogens (Liu et al., 2022b). Taken together, the AsbZIP members of the same subgroup possess similar adjacent phylogenetic relationships, type of conserved domains and motifs, and exon-intron patterns; this may contribute to proteins with similar functions. The exact regulating function of AsbZIPs involved in agarwood tree was unmapped; however, preliminary results indicated that AsbZIPs participated in agarwood formation and plant hormone response (**Figures 7**, **8**), especially for subfamily D of AsbZIPs.

### 4.3 Potential roles of AsbZIPs involved in regulating chromone synthesis

Chromones and sesquiterpenes are the principal constituents of agarwood (Li et al., 2021b). Chromones and their derivatives have various important biological activities and contribute to the balsamic, long-acting, characteristically pleasant fragrance of agarwood (Naef, 2011; Li et al., 2021b). The abundance of PECs is an important indicator of high-quality agarwood. Dynamic changes of the predicted precursors and chromones during agarwood formation indicated that type III PKSs may be responsible for the biosynthesis of PECs and PKSs also contribute to the biosynthesis of flavonoids, which are high similarity to the backbone structures of chromones (Liao et al., 2018). Type III PKSs were the key enzymes in the formation of the C6–C5–C6 scaffold of chromones and in chromone biosynthesis (Li et al., 2021b; Wang et al., 2022d). Transcription factors regulate the synthesis of primary and special secondary metabolites in plants. For example, the TFs of *ERF*, *MYC*, *bHLH*, and *WRKY* could regulate sesquiterpene biosynthesis as the positive or negative regulator in *A. sinensis* (Li et al., 2021a). Nevertheless, the regulatory role of TFs involved in chromone biosynthesis is unknown.

Agarwood is the resin mixed with diverse secondary metabolites produced by wounded agarwood trees (Rasool and Mohamed, 2016; Naziz et al., 2019). Plant hormones directly or indirectly regulate signaling networks by inducing or repressing defense genes; this results in plants adapting to a wide range of environmental stresses. Ethylene is an essential and ubiquitous plant hormone. Plants typically increase ET levels when they suffer from various environmental stresses and ET always elicits plant defense responses (Fatma et al., 2022). Ethylene and jasmonic acid are essential signaling molecules for wound-induced activation of secondary metabolism by modulating reactive oxygen species levels in carrot tissue (Jacobo-Velázquez et al., 2015); ethylene is also a vital signaling molecule in the synthesis of ginsenoside and catechins (Rahimi et al., 2015; Ke et al., 2018). The cultured shoots of agarwood trees treated with MeJA promote the production of sesquiterpene and a chromone derivative of agarwood in *A. sinensis* (Faizal et al., 2021). 2-(2-phenethyl) chromones and their derivatives were induced by various exogenous phytohormones in the calli of agarwood tree, such as MeJA, SA, and abscisic acid (Dong et al., 2018). The involvement of subfamily D bZIPs in plant response to hormone and pathogen infection implied that AsbZIPs might also participate in the defense response of *A. sinensis* resulting in agarwood formation (Mohamed et al., 2010; Chhipa et al., 2017). Two *AsbZIP* genes of subfamily D (*AsbZIP14* and *AsbZIP41*) were highly expressed and significantly downregulated following treatment with a liquid mixture used in agarwood formation (**Figure 8**). This suggested that *AsbZIP14* and *AsbZIP41* might participate in agarwood formation. Ethylene responds to bZIP factors in group D to active pathogen defense and it also is a signaling molecule that induces the expression of subgroup D *bZIP* genes (Tucker et al., 2002; Zander et al., 2014). Furthermore, *AsbZIP14* expression was induced 71,427.18 times following ET treatment, while the expression of other subgroup D*AsbZIP*s have little change according to qRT-PCR assays (**Figure 8A**). These results suggested that *ASbZIP14* and *AsbZIP41* may be the candidate genes for *Aquilaria* trees responding to pathogen challenge and regulating the production of key components in agarwood.

bZIP transcription factors could activate chalcone synthase (CHS), a kind of plant type III PKS involved in flavonoid biosynthesis and plant development or plant signaling transduction in response to pathogens and other stresses. The G/HBF-1 (bZIP) in soybean interacts with CHS promoters to improve disease resistance (Dröge-Laser et al., 1997). *GmbZIP45* overexpression specifically activated *CHS8* transcription and plant defense against pathogens in soybean protoplasts (Gonçalves et al., 2020). *HY5* (bZIPs of subgroup H), a light, and a UV-B radiation response factor acted as a transcriptional activator of *CHS* participating in flavonoid accumulation in *A. thaliana* (Stracke et al., 2010). VvibZIPC22 activates the promoters of the *CHS* gene and controls flavonol biosynthesis (Malacarne et al., 2016). Some type III PKS are involved in chromones or their precursor; however, the roles of *bZIP* in chromone biosynthesis via regulating type III *PKS* expression is poorly studied. This study determined whether *AsbZIP14* and *AsbZIP41* from subfamily D bind with the promoters of *AsPKS3*, *AsPKS4*, *AsPKS6*, *AsPKS8*, and *AsPKS9*. *AsbZIP14* and *AsbZIP41* are a pair of paralogous subfamily D *AsbZIP* genes sharing the most adjacent phylogenetic relationship and having the same number and type of conserved domains and motifs (**Figure 6**). They regulate *AsPKS* expression in different manners, and both were generated from WGD (**Figure 4**). *AsbZIP14* positively activated the expression of *AsPKS3*, *AsPKS6*, and *AsPKS8*, and negatively regulated *AsPKS9* expression. *AsbZIP14* did not interact with the promoters of *AsPKS4*. Interestingly, *AsbZIP41* positively regulated *AsPKS4* by binding its promoters, but cannot activate *AsPKS3*, *AsPKS6*, *AsPKS8*, and *AsPKS9* (**Figure 9D**). The results furtherly indicated that paralogous AsbZIPs from the same subgroup with high sequence homology regulate different target-genes of the same family by varying degrees. This results also demonstrated that bZIPs regulate type III *PKS* expression to promote or suppress flavonoid and chromone generation.

## 5 Conclusion

Systemic genome-wide analysis of 1150 *bZIPs* identified from eight Mavales and three model species indicated that *bZIPs* are an ancient and conserved gene family in the plant kingdom and the recent WGD is the driving force in the evolution of the *bZIP* gene family. The *bZIP* gene family in *A. sinensis* underwent purifying selection in species generation and WGD or segmental duplication resulted in *AsbZIP* expansion. Sixty-two *AsbZIPs* were divided into 13 subfamilies and showed diverse expression profiles during agarwood formation. *AsbZIP14* and *AsbZIP41* from subgroup D had the closest phylogenetic relationship and responded to ET stimulation and agarwood inducer. *AsbZIP14* could interacted with the promoters of *AsPKS3*, *AsPKS6*, *AsPKS8* and positively regulated their expression. Meanwhile, *AsbZIP14* could negatively regulate *AsPKS9* expression. *AsbZIP41* negatively regulated *AsPKS4* expression and could not interact with the other four *AsPKS*. This genome-wide analysis of the *bZIP* gene family provided a basis to further investigate the evolutionary process of *bZIP*s in the plant kingdom and their regulatory mechanisms of type III *AsPKS* in *A. sinensis*. Furthermore, our study also provided potential genetic resources for improving the yield and quantity of agarwood.

## Data availability statement

The datasets presented in this study can be found in online repositories. The names of the repository/repositories and accession number(s) can be found in the article/**Supplementary Material.**

## Author contributions

XD and WM conceived the experiments. HZ and XD carried out the experiments with the help of HW, HC, WD, JZ, and HD. SP and JW contributed the plant materials and data analysis. HZ and XD wrote the manuscript and WM edited the manuscript. All authors contributed to the article and approved the submitted version.

## Supplementary material

Supplementary Figure 1 Motif logos of AsbZIPs.

Supplementary Figure 2 Analysis of *cis* elements in the promoters of *AsbZIP* genes.

Supplementary Figure 3 COG enrichment and classification of *AsbZIPs* and their nearby genes within gene clusters.

Supplementary Figure 4 Gene duplicated *AsbZIPs* and their nearby genes within gene clusters.

## References

[B1] Agarwal P. Baranwal V. K. Khurana P. (2019). Genome-wide analysis of bZIP transcription factors in wheat and functional characterization of a TabZIP under abiotic stress. Sci. Rep. 9 (1), 1–18. doi: 10.1038/s41598-019-40659-7 30872683 PMC6418127

[B2] An J. P. Yao J. F. Xu R. R. You C. X. Wang X. F. Hao Y. J. (2018). Apple bZIP transcription factor MdbZIP44 regulates abscisic acid-promoted anthocyanin accumulation. Plant Cell Environ. 41 (11), 2678–2692. doi: 10.1111/pce.13393 29940702

[B3] Argout X. Salse J. Aury J.-M. Guiltinan M. J. Droc G. Gouzy J.. (2011). The genome of *Theobroma cacao* . Nat. Genet. 43 (2), 101–108. doi: 10.1038/ng.736 21186351

[B4] Bailey T. L. Boden M. Buske F. A. Frith M. Grant C. E. Clementi L.. (2009). MEME SUITE: tools for motif discovery and searching. Nucleic Acids Res. 37 (suppl_2), W202–W208. doi: 10.1093/nar/gkp335 19458158 PMC2703892

[B5] Banerjee A. Roychoudhury A. (2017). Abscisic-acid-dependent basic leucine zipper (bZIP) transcription factors in plant abiotic stress. Protoplasma 254 (1), 3–16. doi: 10.1007/s00709-015-0920-4 26669319

[B6] Broun P. (2004). Transcription factors as tools for metabolic engineering in plants. Curr. Opin. Plant Biol. 7 (2), 202–209. doi: 10.1016/j.pbi.2004.01.013 15003222

[B7] Canaguier A. Grimplet J. Di Gaspero G. Scalabrin S. Duchêne E. Choisne N.. (2017). A new version of the grapevine reference genome assembly (12X. v2) and of its annotation (VCost. v3). Genomics Data 14, 56. doi: 10.1016/j.gdata.2017.09.002 28971018 PMC5612791

[B8] Chhipa H. Chowdhary K. Kaushik N. (2017). Artificial production of agarwood oil in Aquilaria sp. by fungi: a review. Phytochem. Rev. 16 (5), 835–860. doi: 10.1007/s11101-017-9492-6

[B9] Choi J. Huh S. U. Kojima M. Sakakibara H. Paek K.-H. Hwang I. (2010). The cytokinin-activated transcription factor ARR2 promotes plant immunity via TGA3/NPR1-dependent salicylic acid signaling in *Arabidopsis* . Dev. Cell 19 (2), 284–295. doi: 10.1016/j.devcel.2010.07.011 20708590

[B10] Collin A. Daszkowska-Golec A. Kurowska M. Szarejko I. (2020). Barley ABI5 (Abscisic Acid INSENSITIVE 5) is involved in abscisic acid-dependent drought response. Front. Plant Sci. 11. doi: 10.3389/fpls.2020.01138 PMC7405899 32849699

[B11] Corrêa L. G. G. Riaño-Pachón D. M. Schrago C. G. Vicentini dos Santos R. Mueller-Roeber B. Vincentz M. (2008). The role of bZIP transcription factors in green plant evolution: adaptive features emerging from four founder genes. PloS One 3 (8), e2944. doi: 10.1371/journal.pone.0002944 18698409 PMC2492810

[B12] Ding X. Mei W. Huang S. Wang H. Zhu J. Hu W.. (2018). Genome survey sequencing for the characterization of genetic background of *Dracaena Cambodiana* and its defense response during dragon’s blood formation. PloS One 13, e0209258. doi: 10.1371/journal.pone.0209258 30550595 PMC6294377

[B13] Ding X. Mei W. Lin Q. Wang H. Wang J. Peng S.. (2020). Genome sequence of the agarwood tree *Aquilaria sinensis* (Lour.) Spreng: the first chromosome-level draft genome in the Thymelaeceae family. GigaScience 9 (3), giaa013. doi: 10.1093/gigascience/giaa013 32118265 PMC7050300

[B14] Dong X. Gao B. Feng Y. Liu X. Wang J. Wang J.. (2018). Production of 2-(2-phenylethyl) chromones in *Aquilaria sinensis* calli under different treatments. Plant Cell Tiss. Org. 135 (1), 53–62. doi: 10.1007/s11240-018-1442-5

[B15] Dröge-Laser W. Kaiser A. Lindsay W. P. Halkier B. A. Loake G. J. Doerner P.. (1997). Rapid stimulation of a soybean protein-serine kinase that phosphorylates a novel bZIP DNA-binding protein, G/HBF-1, during the induction of early transcription-dependent defenses. EMBO J. 16 (4), 726–738. doi: 10.1093/emboj/16.4.726 9049302 PMC1169674

[B16] Dröge-Laser W. Snoek B. L. Snel B. Weiste C. (2018). The *Arabidopsis* bZIP transcription factor family—an update. Curr. Opin. Plant Biol. 45, 36–49. doi: 10.1016/j.pbi.2018.05.001 29860175

[B17] Duan L. Mo Z. Fan Y. Li K. Yang M. Li D.. (2022). Genome-wide identification and expression analysis of the bZIP transcription factor family genes in response to abiotic stress in *Nicotiana tabacum* L. BMC Genomics 23 (1), 1–17. doi: 10.1186/s12864-022-08547-z 35448973 PMC9027840

[B18] Faizal A. Esyanti R. R. Adn’ain N. Rahmani S. Azar A. W. P. Turjaman M. (2021). Methyl jasmonate and crude extracts of *Fusarium solani* elicit agarwood compounds in shoot culture of *Aquilaria malaccensis* Lamk. Heliyon 7 (4), e06725. doi: 10.1016/j.heliyon.2021.e06725 33948505 PMC8080053

[B19] Fatma M. Asgher M. Iqbal N. Rasheed F. Sehar Z. Sofo A.. (2022). Ethylene signaling under stressful environments: Analyzing collaborative knowledge. Plants 11 (17), 2211. doi: 10.3390/plants11172211 36079592 PMC9460115

[B20] Fonseca A. Urzúa T. Jelenska J. Sbarbaro C. Seguel A. Duarte Y.. (2022). The TGA transcription factors from clade II negatively regulate the salicylic acid accumulation in *Arabidopsis* . Int. J. Mol. Scl. 23 (19), 11631. doi: 10.3390/ijms231911631 PMC9569720 36232932

[B21] Foster R. Izawa T. Chua N. H. (1994). Plant bZIP proteins gather at ACGT elements. FASEB J. 8 (2), 192–200. doi: 10.1096/fasebj.8.2.8119490 8119490

[B22] Fu Z. Q. Dong X. (2013). Systemic acquired resistance: turning local infection into global defense. Annu. Rev. Plant Biol. 64, 839–863. doi: 10.1146/annurev-arplant-042811-105606 23373699

[B23] Gasteiger E. Gattiker A. Hoogland C. Ivanyi I. Appel R. D. Bairoch A. (2003). ExPASy: the proteomics server for in-depth protein knowledge and analysis. Nucleic Acids Res. 31 13), 3784–3788. doi: 10.1093/nar/gkg563 PMC168970 12824418

[B24] Goll J. Rusch D. B. Tanenbaum D. M. Thiagarajan M. Li K. Methé B. A.. (2010). METAREP: JCVI metagenomics reports—an open source tool for high-performance comparative metagenomics. Bioinformatics 26 (20), 2631–2632. doi: 10.1093/bioinformatics/btq455 20798169 PMC2951084

[B25] Gonçalves A. B. Fontes P. P. Dadalto S. P. de Souza G. B. Marcelino-Guimarães F. C. Alves M. S.. (2020). GmbZIP45 binds to H-box cis-element *in vitro* and overexpression in soybean protoplasts induces the expression of *CHS8* gene. Physiol. Mol. Plant P. 112, 101556. doi: 10.1016/j.pmpp.2020.101556

[B26] Guan X. Nah G. Song Q. Udall J. A. Stelly D. M. Chen Z. J. (2014). Transcriptome analysis of extant cotton progenitors revealed tetraploidization and identified genome-specific single nucleotide polymorphism in diploid and allotetraploid cotton. BMC Res. Notes 7 (1), 1–10. doi: 10.1186/1756-0500-7-493 25099166 PMC4267057

[B27] Guan R. Xu S. Lu Z. Su L. Zhang L. Sun W.. (2022). Genomic characterization of bZIP transcription factors related to andrographolide biosynthesis in *Andrographis paniculata* . Int. J. Biol. Macromol. 223, 1619–1631. doi: 10.1016/j.ijbiomac.2022.10.283 36356875

[B28] Hao X. Zhong Y. Nützmann H.-W. Fu X. Yan T. Shen Q.. (2019). Light-induced artemisinin biosynthesis is regulated by the bZIP transcription factor AaHY5 in *Artemisia annua* . Plant Cell Physiol. 60 (8), 1747–1760. doi: 10.1093/pcp/pcz084 31076768

[B29] Hou H. Kong X. Zhou Y. Yin C. Jiang Y. Qu H.. (2022). Genome-wide identification and characterization of bZIP transcription factors in relation to litchi (*Litchi chinensis* Sonn.) fruit ripening and postharvest storage. Int. J. Biol. Macromol. 222, 2176–2189. doi: 10.1016/j.ijbiomac.2022.09.292 36208810

[B30] Hu B. Jin J. Guo A.-Y. Zhang H. Luo J. Gao G. (2015). GSDS 2.0: an upgraded gene feature visualization server. Bioinformatics 31 (8), 1296–1297. doi: 10.1093/bioinformatics/btu817 25504850 PMC4393523

[B31] Hu W. Yang H. Yan Y. Wei Y. Tie W. Ding Z.. (2016). Genome-wide characterization and analysis of *bZIP* transcription factor gene family related to abiotic stress in cassava. Sci. Rep. 6 (1), 1–12. doi: 10.1038/srep22783 26947924 PMC4780028

[B32] Ishihara M. Tsuneya T. Uneyama K. (1993). Components of the volatile concentrate of agarwood. J. Essent. Oil Res. 5 (3), 283–289. doi: 10.1080/10412905.1993.9698221

[B33] Jacobo-Velázquez D. A. González-Agüero M. Cisneros-Zevallos L. (2015). Cross-talk between signaling pathways: the link between plant secondary metabolite production and wounding stress response. Sci. Rep. 5 (1), 1–10. doi: 10.1038/srep08608 PMC5390084 25712739

[B34] Jakoby M. Weisshaar B. Dröge-Laser W. Vicente-Carbajosa J. Tiedemann J. Kroj T.. (2002). bZIP transcription factors in *Arabidopsis* . Trends Plant Sci. 7 (3), 106–111. doi: 10.1016/S1360-1385(01)02223-3 11906833

[B35] Jiang M. Wang Z. Ren W. Yan S. Xing N. Zhang Z.. (2022). Identification of the *bZIP* gene family and regulation of metabolites under salt stress in *lsatis indigotica* . Front. Plant Sci. 13. doi: 10.3389/fpls.2022.1011616 PMC9576947 36267941

[B36] Job N. Yadukrishnan P. Bursch K. Datta S. Johansson H. (2018). Two B-box proteins regulate photomorphogenesis by oppositely modulating HY5 through their diverse C-terminal domains. Plant Physiol. 176 (4), 2963–2976. doi: 10.1104/pp.17.00856 29439209 PMC5884587

[B37] Ke S.-W. Chen G.-H. Chen C.-T. Tzen J. T. Yang C.-Y. (2018). Ethylene signaling modulates contents of catechin and ability of antioxidant in *Camellia sinensis* . Bot. Stud. 59 (1), 1–8. doi: 10.1186/s40529-018-0226-x 29616373 PMC5882471

[B38] Kim J.-S. Yamaguchi-Shinozaki K. Shinozaki K. (2018). ER-anchored transcription factors bZIP17 and bZIP28 regulate root elongation. Plant Physiol. 176 (3), 2221–2230. doi: 10.1104/pp.17.01414 29367234 PMC5841724

[B39] Koch M. A. Haubold B. Mitchell-Olds T. (2000). Comparative evolutionary analysis of chalcone synthase and alcohol dehydrogenase loci in *Arabidopsis*, *Arabis*, and related genera (Brassicaceae). Mol. Biol. Evol. 17 (10), 1483–1498. doi: 10.1093/oxfordjournals.molbev.a026248 11018155

[B40] Kouzarides T. Ziff E. (1989). Leucine zippers of fos, jun and GCN4 dictate dimerization specificity and thereby control DNA binding. Nature 340 (6243), 568–571. doi: 10.1038/340568a0 2505081

[B41] Krzywinski M. Schein J. Birol I. Connors J. Gascoyne R. Horsman D.. (2009). Circos: an information aesthetic for comparative genomics. Genome Res. 19 (9), 1639–1645. doi: 10.1101/gr.092759.109 19541911 PMC2752132

[B42] Kumeta Y. Ito M. (2010). Characterization of δ-guaiene synthases from cultured cells of *Aquilaria*, responsible for the formation of the sesquiterpenes in agarwood. Plant Physiol. 154 (4), 1998–2007. doi: 10.1104/pp.110.161828 20959422 PMC2996018

[B43] Lescot M. Déhais P. Thijs G. Marchal K. Moreau Y. Van de Peer Y.. (2002). PlantCARE, a database of plant cis-acting regulatory elements and a portal to tools for *in silico* analysis of promoter sequences. Nucleic Acids Res. 30 (1), 325–327. doi: 10.1093/nar/30.1.325 11752327 PMC99092

[B44] Li W. Chen H.-Q. Wang H. Mei W.-L. Dai H.-F. (2021b). Natural products in agarwood and *Aquilaria* plants: chemistry, biological activities and biosynthesis. Nat. Prod. Rep. 38 (3), 528–565. doi: 10.1039/D0NP00042F 32990292

[B45] Li H. Li L. ShangGuan G. Jia C. Deng S. NOman M.. (2020). Genome-wide identification and expression analysis of *bZIP* gene family in *Carthamus tinctorius* L. Sci. Rep. 10 (1), 1–15. doi: 10.1038/s41598-020-72390-z 32968100 PMC7511407

[B46] Li R.-S. Zhu J.-H. Guo D. Li H.-L. Wang Y. Ding X.-P.. (2021a). Genome-wide identification and expression analysis of terpene synthase gene family in *Aquilaria sinensis* . Plant Physiol. Bioch. 164, 185–194. doi: 10.1016/j.plaphy.2021.04.028 34004556

[B47] Lian H. Xu P. He S. Wu J. Pan J. Wang W.. (2018). Photoexcited CRYPTOCHROME 1 interacts directly with G-protein β subunit AGB1 to regulate the DNA-binding activity of HY5 and photomorphogenesis in *Arabidopsis* . Mol. Plant 11 (10), 1248–1263. doi: 10.1016/j.molp.2018.08.004 30176372

[B48] Liang Y.-E. Zhang H. Zhu J. Wang H. Mei W. Jiang B.. (2023). Transcriptomic analysis reveals the involvement of flavonoids synthesis genes and transcription factors in *dracaena Cambodiana* response to ultraviolet-B radiation. Forests 14 (5), 979. doi: 10.3390/f14050979

[B49] Liao G. Dong W.-H. Yang J.-L. Li W. Wang J. Mei W.-L.. (2018). Monitoring the chemical profile in agarwood formation within one year and speculating on the biosynthesis of 2-(2-phenylethyl) chromones. Molecules 23 (6), 1261. doi: 10.3390/molecules23061261 29799457 PMC6100365

[B50] Liu J. Li T. Chen T. Gao J. Zhang X. Jiang C.. (2022a). Integrating Multiple Omics Identifies *Phaeoacremonium rubrigenum* acting as *Aquilaria sinensis* marker fungus to promote agarwood sesquiterpene accumulation by inducing plant host phosphorylation. Microbiol. Spectr. 10 (4), e02722–e02721. doi: 10.1128/spectrum.02722-21 35762771 PMC9431625

[B51] Liu W. Zhao C. Liu L. Huang D. Ma C. Li R.. (2022b). Genome-wide identification of the TGA gene family in kiwifruit (*Actinidia chinensis* spp.) and revealing its roles in response to *Pseudomonas syringae* pv. *actinidiae* (Psa) infection. Int. J. Biol. Macromol. 222, 101–113. doi: 10.1016/j.ijbiomac.2022.09.154 36150565

[B52] Loyola R. Herrera D. Mas A. Wong D. C. J. Höll J. Cavallini E.. (2016). The photomorphogenic factors UV-B RECEPTOR 1, ELONGATED HYPOCOTYL 5, and HY5 HOMOLOGUE are part of the UV-B signalling pathway in grapevine and mediate flavonol accumulation in response to the environment. J. Exp. Bot. 67 (18), 5429–5445. doi: 10.1093/jxb/erw307 27543604 PMC5049392

[B53] Lu M. Meng X.-X. Zhang Y.-M. Zhu X.-W. Li J. Chen W.-Q.. (2022). Genome-Wide Identification and Expression Profiles of bZIP Genes in *Cannabis sativa* L. Cannabis Cannabinoid. 7 (6), 882–895. doi: 10.1089/can.2021.0153 35020417

[B54] Ma M. Chen Q. Dong H. Zhang S. Huang X. (2021). Genome-wide identification and expression analysis of the bZIP transcription factors, and functional analysis in response to drought and cold stresses in pear (*Pyrus breschneideri*). BMC Plant Biol. 21 (1), 1–19. doi: 10.1186/s12870-021-03356-0 34886805 PMC8656046

[B55] Malacarne G. Coller E. Czemmel S. Vrhovsek U. Engelen K. Goremykin V.. (2016). The grapevine VvibZIPC22 transcription factor is involved in the regulation of flavonoid biosynthesis. J. Exp. Bot. 67 (11), 3509–3522. doi: 10.1093/jxb/erw181 27194742 PMC4892739

[B56] Meng F. Chu T. Tang Q. Chen W. (2021). A tetraploidization event shaped the *Aquilaria sinensis* genome and contributed to the ability of sesquiterpenes synthesis. BMC Genomics 22 (1), 1–12. doi: 10.1186/s12864-021-07965-9 34493201 PMC8424979

[B57] Minh B. Q. Schmidt H. A. Chernomor O. Schrempf D. Woodhams M. D. Von Haeseler A.. (2020). IQ-TREE 2: new models and efficient methods for phylogenetic inference in the genomic era. Mol. Biol. Evol. 37 (5), 1530–1534. doi: 10.1093/molbev/msaa015 32011700 PMC7182206

[B58] Mohamed R. Jong P. Zali M. (2010). Fungal diversity in wounded stems of *Aquilaria malaccensis* . Fungal Divers. 43 (1), 67–74. doi: 10.1007/s13225-010-0039-z

[B59] Naef R. (2011). The volatile and semi-volatile constituents of agarwood, the infected heartwood of *Aquilaria* species: a review. Flavour Frag. J. 26 (2), 73–87. doi: 10.1002/ffj.2034

[B60] Naziz P. S. Das R. Sen S. (2019). The scent of stress: Evidence from the unique fragrance of agarwood. Front. Plant Sci. 10. doi: 10.3389/fpls.2019.00840 PMC6646531 31379890

[B61] Pedrotti L. Weiste C. Nägele T. Wolf E. Lorenzin F. Dietrich K.. (2018). Snf1-RELATED KINASE1-controlled C/S1-bZIP signaling activates alternative mitochondrial metabolic pathways to ensure plant survival in extended darkness. Plant Cell 30 (2), 495–509. doi: 10.1105/tpc.17.00414 29348240 PMC5868691

[B62] Persoon G. A. (2008). “Growing ‘the wood of the gods’: agarwood production in Southeast Asia”. in Smallholder tree growing for Rural Development and Environmental Services. Eds. Snelder D. J. Lasco R. D. (Dordrecht, NL: Springer Press), 245–262. doi: 10.1007/978-1-4020-8261-0_12

[B63] Project A. G. Albert V. A. Barbazuk W. B. dePamphilis C. W. Der J. P. Leebens-Mack J.. (2013). The *Amborella* genome and the evolution of flowering plants. Science 342 (6165), 1241089. doi: 10.1126/science.1241089 24357323

[B64] Qu L. Li H.-L. Guo D. Wang Y. Zhu J.-H. Yin L.-Y.. (2020). HbWRKY27, a group IIe WRKY transcription factor, positively regulates *HbFPS1* expression in *Hevea brasiliensis* . Sci. Rep. 10 (1), 1–8. doi: 10.1038/s41598-020-77805-5 33244131 PMC7692525

[B65] Qu D. Wu F. Zhao X. Zhu D. Gu L. Yang L.. (2022). A bZIP transcription factor VabZIP12 from blueberry induced by dark septate endocyte improving the salt tolerance of transgenic *Arabidopsis* . Plant Sci. 315, 111135. doi: 10.1016/j.plantsci.2021.111135 35067305

[B66] Rahimi S. Kim Y.-J. Yang D.-C. (2015). Production of ginseng saponins: elicitation strategy and signal transductions. Appl. Microbiol. Biot. 99 (17), 6987–6996. doi: 10.1007/s00253-015-6806-8 26194557

[B67] Rasool S. Mohamed R. (2016). “Understanding agarwood formation and its challenges. in: Agarwood science behind the fragrance” Ed. Mohamed R. (Singapore, SG: Springer Press), 39–56. doi: 10.1007/978-981-10-0833-7_3

[B68] Rong S. Wu Z. Cheng Z. Zhang S. Liu H. Huang Q. (2020). Genome-wide identification, evolutionary patterns, and expression analysis of *bZIP* gene family in olive (*Olea europaea* L.). Genes 11 (5), 510. doi: 10.3390/genes11050510 32380769 PMC7288668

[B69] Ruberti C. Lai Y. Brandizzi F. (2018). Recovery from temporary endoplasmic reticulum stress in plants relies on the tissue-specific and largely independent roles of bZIP28 and bZIP60, as well as an antagonizing function of BAX-Inhibitor 1 upon the pro-adaptive signaling mediated by bZIP28. Plant J. 93 (1), 155–165. doi: 10.1111/tpj.13768 29124827 PMC5732024

[B70] Sangareswari M. Parthiban K. T. Kanna S. U. Karthiba L. Saravanakumar D. (2016). Fungal microbes associated with agarwood formation. Am. J. Plant Sci. 7 (10), 1445–1452. doi: 10.4236/ajps.2016.710138

[B71] Shine M. Xiao X. Kachroo P. Kachroo A. (2019). Signaling mechanisms underlying systemic acquired resistance to microbial pathogens. Plant Sci. 279, 81–86. doi: 10.1016/j.plantsci.2018.01.001 30709496

[B72] Shu K. Chen F. Zhou W. Luo X. Dai Y. Shuai H.. (2018). ABI4 regulates the floral transition independently of ABI5 and ABI3. Mol. Biol. Rep. 45 (6), 2727–2731. doi: 10.1007/s11033-018-4290-9 30121823

[B73] Song X. Ma X. Li C. Hu J. Yang Q. Wang T. (2018). Comprehensive analyses of the *BES1* gene family in *Brassica napus* and examination of their evolutionary pattern in representative species. BMC Genomics 19 (1), 1–15. doi: 10.1186/s12864-018-4744-4 29743014 PMC5944053

[B74] Stracke R. FAVORY J. J. Gruber H. Bartelniewoehner L. Bartels S. Binkert M.. (2010). The *Arabidopsis* bZIP transcription factor HY5 regulates expression of the *PFG1*/*MYB12* gene in response to light and ultraviolet-B radiation. Plant Cell Environ. 33 (1), 88–103. doi: 10.1111/j.1365-3040.2009.02061.x 19895401

[B75] Sun P. Jiao B. Yang Y. Shan L. Li T. Li X.. (2022b). WGDI: A user-friendly toolkit for evolutionary analyses of whole-genome duplications and ancestral karyotypes. Mol. Plant 15 (12), 1841–1851. doi: 10.1016/j.molp.2022.10.018 36307977

[B76] Tucker M. L. Whitelaw C. A. Lyssenko N. N. Nath P. (2002). Functional analysis of regulatory elements in the gene promoter for an abscission-specific cellulase from bean and isolation, expression, and binding affinity of three TGA-type basic leucine zipper transcription factors. Plant Physiol. 130 (3), 1487–1496. doi: 10.1104/pp.007971 12428013 PMC166667

[B77] Unel N. M. Cetin F. Karaca Y. Celik Altunoglu Y. Baloglu M. C. (2019). Comparative identification, characterization, and expression analysis of *bZIP* gene family members in watermelon and melon genomes. Plant Growth Regul. 87 (2), 227–243. doi: 10.1007/s10725-018-0465-6

[B78] Vanneste K. Baele G. Maere S. Van de Peer Y. (2014). Analysis of 41 plant genomes supports a wave of successful genome duplications in association with the Cretaceous–Paleogene boundary. Genome Res. 24 (8), 1334–1347. doi: 10.1101/gr.168997.113 24835588 PMC4120086

[B79] Vinson C. R. Sigler P. B. McKnight S. L. (1989). Scissors-grip model for DNA recognition by a family of leucine zipper proteins. Science 246 (4932), 911–916. doi: 10.1126/science.2683088 2683088

[B80] Wang L. Fobert P. R. (2013). Arabidopsis clade I TGA factors regulate apoplastic defences against the bacterial pathogen *Pseudomonas syringae* through endoplasmic reticulum-based processes. PloS One 8 (9), e77378. doi: 10.1371/journal.pone.0077378 24086773 PMC3785447

[B81] Wang X.-H. Gao B.-W. Nakashima Y. Mori T. Zhang Z.-X. Kodama T.. (2022d). Identification of a diarylpentanoid-producing polyketide synthase revealing an unusual biosynthetic pathway of 2-(2-phenylethyl) chromones in agarwood. Nat. Commun. 13 (1), 1–12. doi: 10.1038/s41467-022-27971-z 35039506 PMC8764113

[B82] Wang S. Liang H. Wang H. Li L. Xu Y. Liu Y.. (2022b). The chromosome-scale genomes of *Dipterocarpus turbinatus* and *Hopea hainanensis* (Dipterocarpaceae) provide insights into fragrant oleoresin biosynthesis and hardwood formation. Plant Biotechnol. J. 20 (3), 538–553. doi: 10.1111/pbi.13735 34687252 PMC8882806

[B83] Wang J. Yuan J. Yu J. Meng F. Sun P. Li Y.. (2019). Recursive Paleohexaploidization shaped the durian genome. Plant Physiol. 179 (1), 209–219. doi: 10.1104/pp.18.00921 30385647 PMC6324235

[B84] Wang S. Zhang X. Li B. Zhao X. Shen Y. Yuan Z. (2022c). Genome-wide identification and characterization of *bZIP* gene family and cloning of candidate genes for anthocyanin biosynthesis in pomegranate (*Punica granatum*). BMC Plant Biol. 22 (1), 1–18. doi: 10.1186/s12870-022-03560-6 35379169 PMC8978422

[B85] Wang H. Zhang Y. Norris A. Jiang C.-Z. (2022a). S1-bZIP transcription factors play important roles in the regulation of fruit quality and stress response. Front. Plant Sci. 12. doi: 10.3389/fpls.2021.802802 PMC8795868 35095974

[B86] Wei K. Chen J. Wang Y. Chen Y. Chen S. Lin Y.. (2012). Genome-wide analysis of *bZIP*-encoding genes in maize. DNA Res. 19 (6), 463–476. doi: 10.1093/dnares/dss026 23103471 PMC3514857

[B87] Weirauch M. T. Hughes T. (2011). A catalogue of eukaryotic transcription factor types, their evolutionary origin, and species distribution, A handbook of transcription factors (Springer), 25–73. 10.1007/978-90-481-9069-0_3 21557078

[B88] Weiste C. Pedrotti L. Selvanayagam J. Muralidhara P. Fröschel C. Novák O.. (2017). The *Arabidopsis* bZIP11 transcription factor links low-energy signalling to auxin-mediated control of primary root growth. PloS Genet. 13 (2), e1006607. doi: 10.1371/journal.pgen.1006607 28158182 PMC5315408

[B89] Weltmeier F. Rahmani F. Ehlert A. Dietrich K. Schütze K. Wang X.. (2009). Expression patterns within the *Arabidopsis* C/S1 bZIP transcription factor network: availability of heterodimerization partners controls gene expression during stress response and development. Plant Mol. Biol. 69 (1), 107–119. doi: 10.1007/s11103-008-9410-9 18841482 PMC2709229

[B90] Wu S. Zhu P. Jia B. Yang J. Shen Y. Cai X.. (2018). A Glycine soja group S2 bZIP transcription factor GsbZIP67 conferred bicarbonate alkaline tolerance in *Medicago sativa* . BMC Plant Biol. 18 (1), 1–10. doi: 10.1186/s12870-018-1466-3 30316294 PMC6186066

[B91] Yang Z. Sun J. Chen Y. Zhu P. Zhang L. Wu S.. (2019). Genome-wide identification, structural and gene expression analysis of the bZIP transcription factor family in sweet potato wild relative *Ipomoea trifida* . BMC Genet. 20 (1), 1–18. doi: 10.1186/s12863-019-0743-y 31023242 PMC6482516

[B92] Zander M. La Camera S. Lamotte O. Métraux J. P. Gatz C. (2010). *Arabidopsis thaliana* class-II TGA transcription factors are essential activators of jasmonic acid/ethylene-induced defense responses. Plant J. 61 (2), 200–210. doi: 10.1111/j.1365-313X.2009.04044.x 19832945

[B93] Zander M. Thurow C. Gatz C. (2014). TGA transcription factors activate the salicylic acid-suppressible branch of the ethylene-induced defense program by regulating *ORA59* expression. Plant Physiol. 165 (4), 1671–1683. doi: 10.1104/pp.114.243360 24989234 PMC4119047

[B94] Zhang Z. (2022). KaKs_Calculator 3.0: calculating selective pressure on coding and non-coding sequences. Genomics Proteomics Bioinf. 20 (3), 536–540. doi: 10.1016/j.gpb.2021.12.002 PMC9801026 34990803

[B95] Zhang Y. Gao W. Li H. Wang Y. Li D. Xue C.. (2020b). Genome-wide analysis of the *bZIP* gene family in Chinese jujube (*Ziziphus jujuba* Mill.). BMC Genet. 21 (1), 1–14. doi: 10.1186/s12864-020-06890-7 32664853 PMC7362662

[B96] Zhang M. Liu Y. Shi H. Guo M. Chai M. He Q.. (2018a). Evolutionary and expression analyses of soybean basic Leucine zipper transcription factor family. BMC Genet. 19 (1), 1–14. doi: 10.1186/s12864-018-4511-6 29471787 PMC5824455

[B97] Zhang L. Ma X. Zhang X. Xu Y. Ibrahim A. K. Yao J.. (2021). Reference genomes of the two cultivated jute species. Plant Biotechnol. J. 19 (11), 2235–2248. doi: 10.1111/pbi.13652 34170619 PMC8541789

[B98] Zhang Z. Quan S. Niu J. Guo C. Kang C. Liu J.. (2022b). Genome-Wide identification, classification, expression and duplication analysis of *bZIP* family genes in *Juglans regia* L. Int. J. Mol. Sci. 23 (11), 5961. doi: 10.3390/ijms23115961 35682645 PMC9180593

[B99] Zhang Y. Xu Z. Ji A. Luo H. Song J. (2018b). Genomic survey of *bZIP* transcription factor genes related to tanshinone biosynthesis in *Salvia miltiorrhiza* . Acta Pharm. Sin. B 8 (2), 295–305. doi: 10.1016/j.apsb.2017.09.002 29719790 PMC5925414

[B100] Zhang L. Xu Y. Zhang X. Ma X. Zhang L. Liao Z.. (2020a). The genome of kenaf (*Hibiscus cannabinus* L.) provides insights into bast fibre and leaf shape biogenesis. Plant Biotechnol. J. 18 (8), 1796–1809. doi: 10.1111/pbi.13341 31975524 PMC7336286

[B101] Zhang Q. Zhang W. J. Yin Z. G. Li W. J. Xia C.-Y. Sun H.-Y.. (2022a). Genome-wide identification reveals the potential functions of the bZIP gene family in common bean (*Phaseolus vulgaris*) in response to salt stress during the sprouting stage. J. Plant Growth Regul. 41 (8), 3075–3090. doi: 10.1007/s00344-021-10497-x

[B102] Zhang Y. Zhou J. Wang L. (2014). Mini review roles of the *bZIP* gene family in rice. Genet. Mol. Res. 13 (2), 3025–3036. doi: 10.4238/2014.april.16.11 24782137

[B103] Zhao B. Wang L. Pang S. Jia Z. Wang L. Li W.. (2020). UV-B promotes flavonoid synthesis in *Ginkgo biloba* leaves. Ind. Crops Prod. 151, 112483. doi: 10.1016/j.indcrop.2020.112483

[B104] Zheng C. Santos Muñoz D. Albert V. A. Sankoff D. (2015). Syntenic block overlap multiplicities with a panel of reference genomes provide a signature of ancient polyploidization events. BMC Genomics 16 (10), 1–6. doi: 10.1186/1471-2164-16-S10-S8 26449933 PMC4603330

[B105] Zhou Y. Massonnet M. Sanjak J. S. Cantu D. Gaut B. S. (2017). Evolutionary genomics of grape (*Vitis vinifera* ssp. *vinifera*) domestication. P. Natl. Acad. Sci. 114 (44), 11715–11720. doi: 10.1073/pnas.1709257114 PMC5676911 29042518

[B106] Zuccolo A. Bowers J. E. Estill J. C. Xiong Z. Luo M. Sebastian A.. (2011). A physical map for the *Amborella trichopoda* genome sheds light on the evolution of angiosperm genome structure. Genome Biol. 12, 1–14. doi: 10.1186/gb-2011-12-5-r48 PMC3219971 21619600

