# Supplementary figures and images for: Systematic evolution of bZIP transcription factors in Malvales and functional exploration of AsbZIP14 and AsbZIP41 in Aquilaria sinensis
Source: Front Plant Sci. 2023 Aug 30;14:1243323. doi: 10.3389/fpls.2023.1243323 (PMC10499555; doi:10.3389/fpls.2023.1243323)

motif1

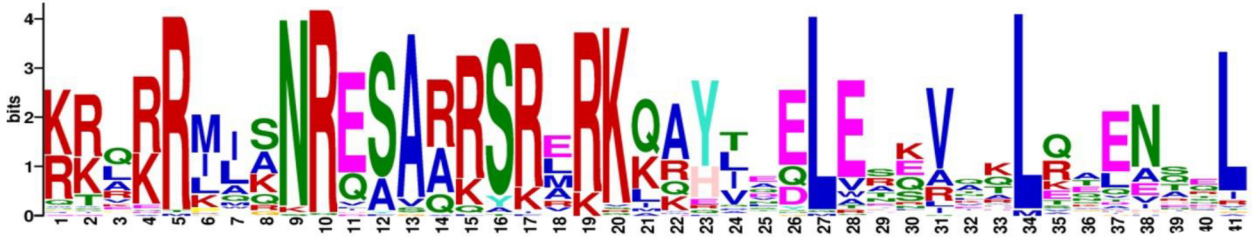

motif2

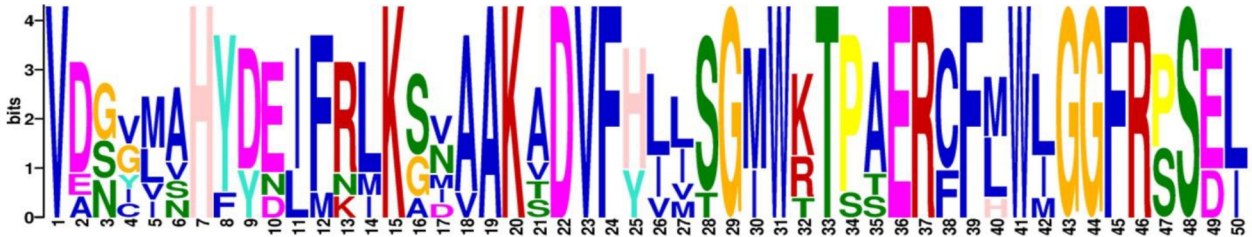

motif3

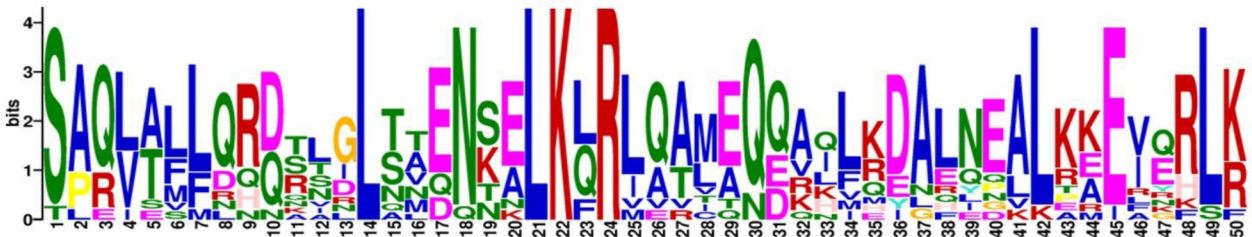

motif4

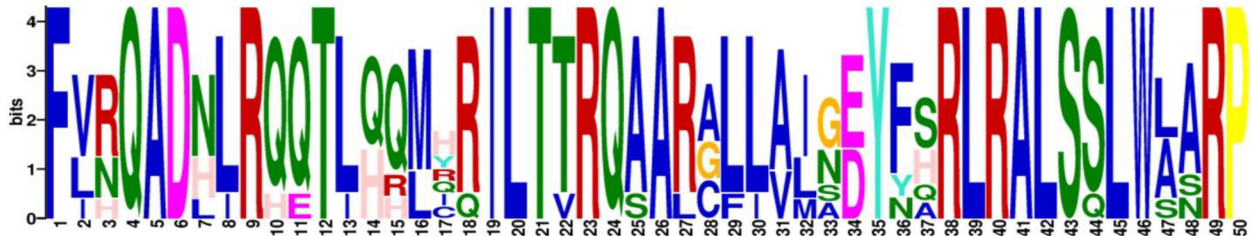

motif5

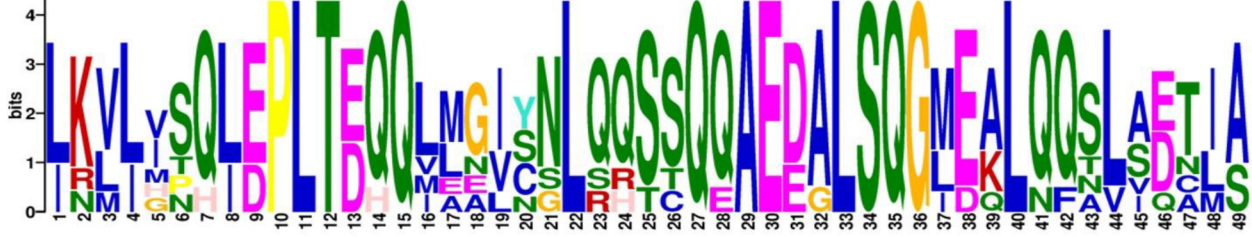

motif6

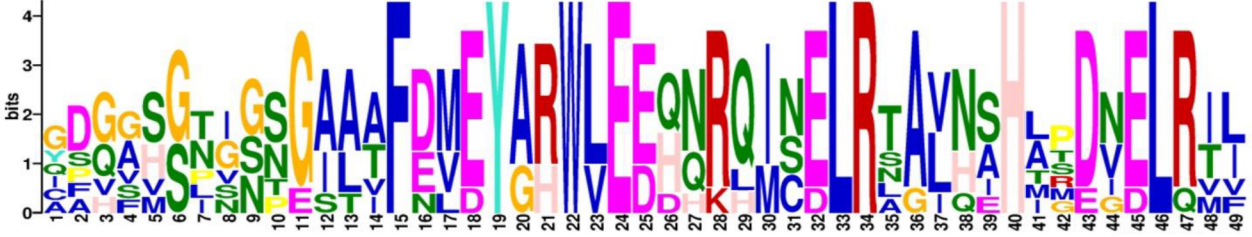

motif7

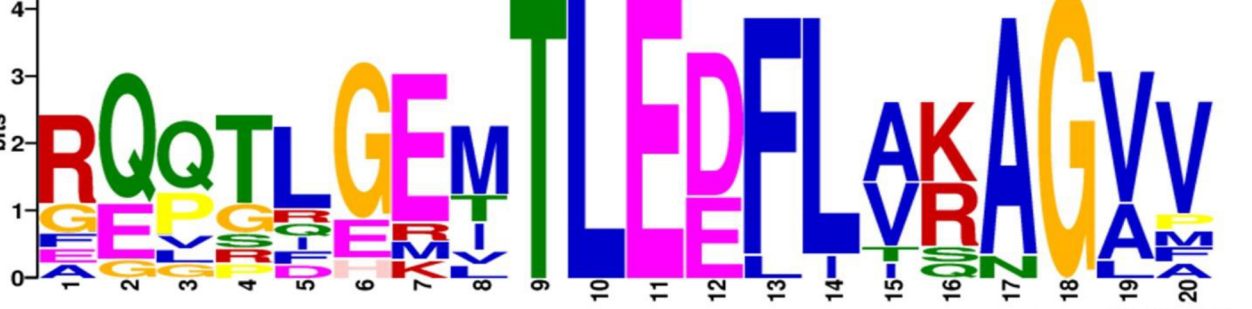

motif8

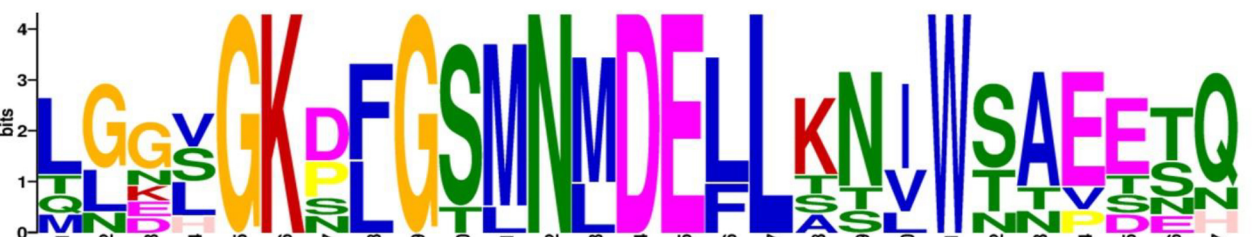

motif9

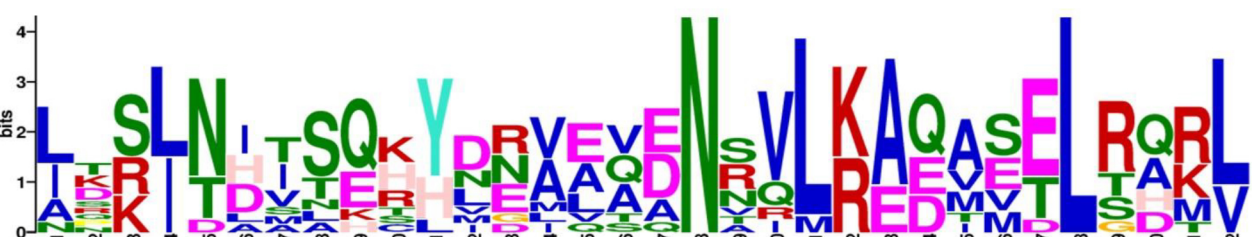

motif10

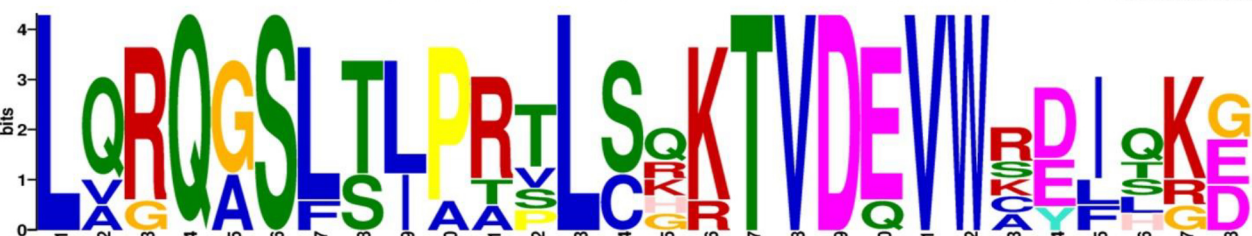

motif11

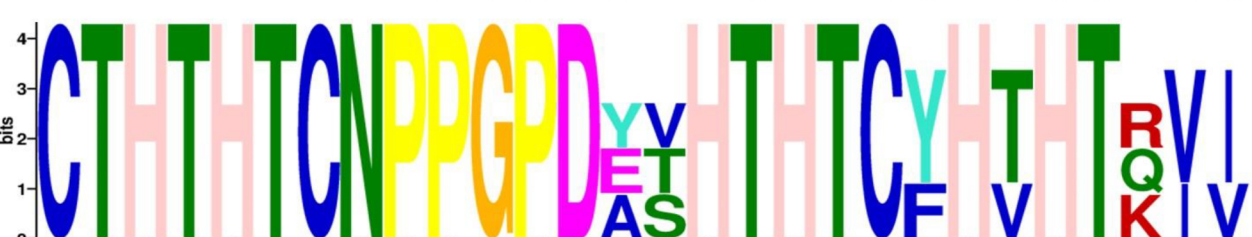

motif12

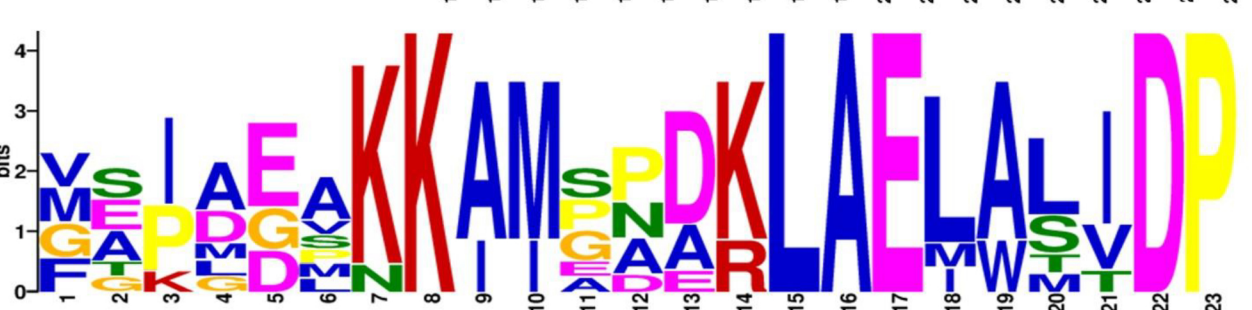

motif13

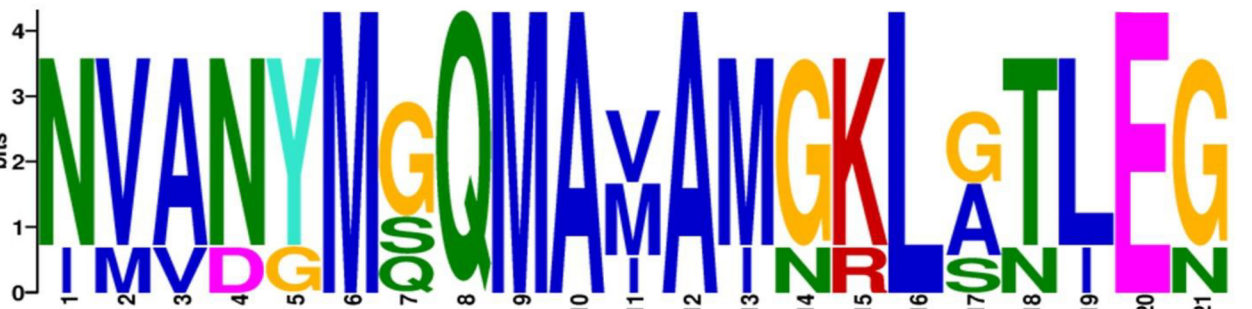

motif14

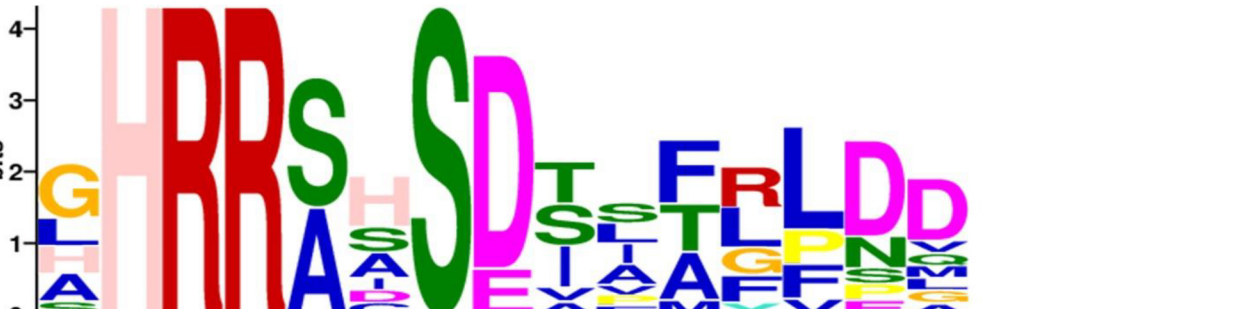

motif15

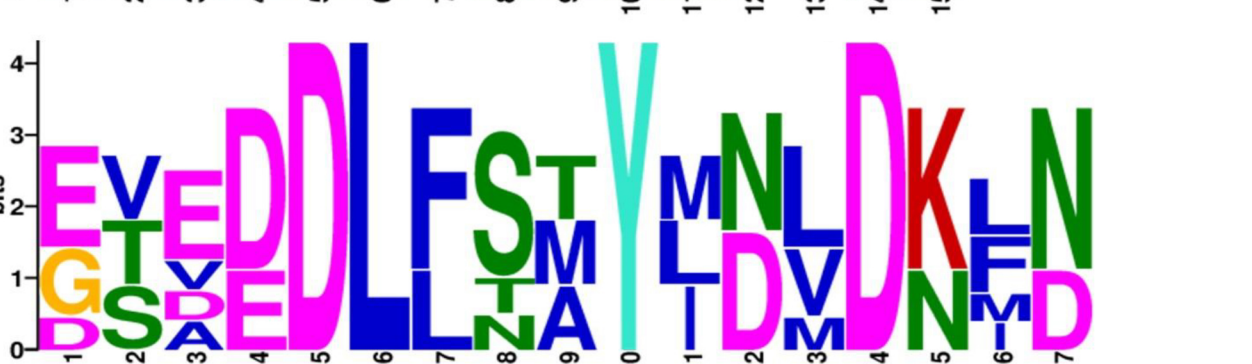

Supplement: Supplementary Figure 1 — Motif logos of AsbZIPs. [file DataSheet_1.pdf]

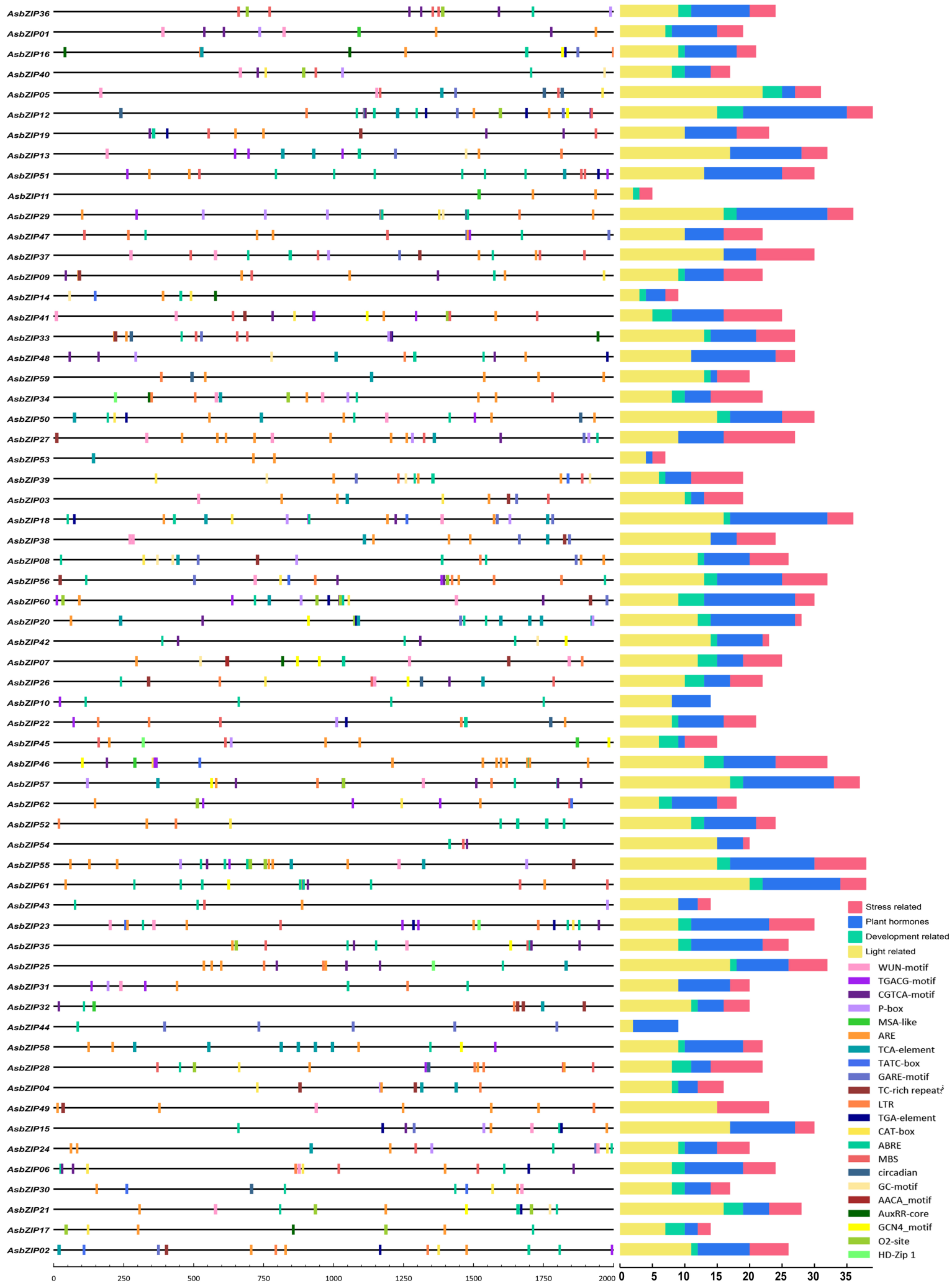

Supplement: Supplementary Figure 2 — Analysis of cis elements in the promoters of AsbZIP genes. [file DataSheet_2.pdf]

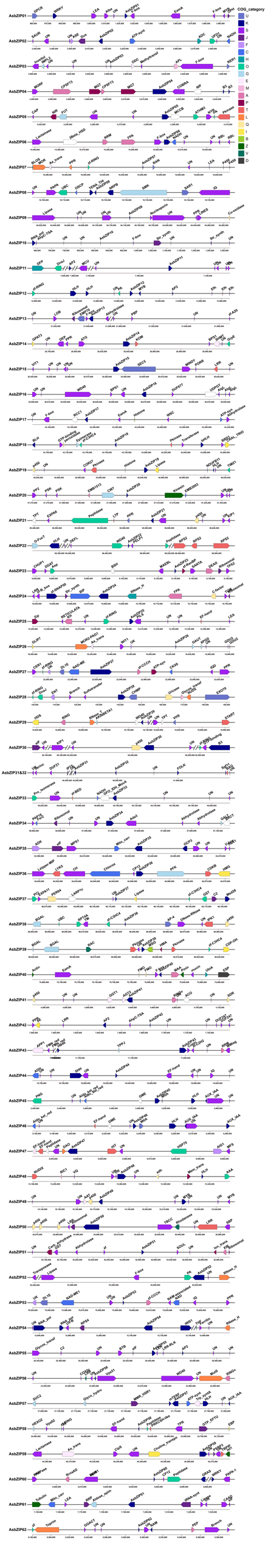

Supplement: Supplementary Figure 3 — COG enrichment and classification of AsbZIPs and their nearby genes within gene clusters. [file DataSheet_3.pdf]

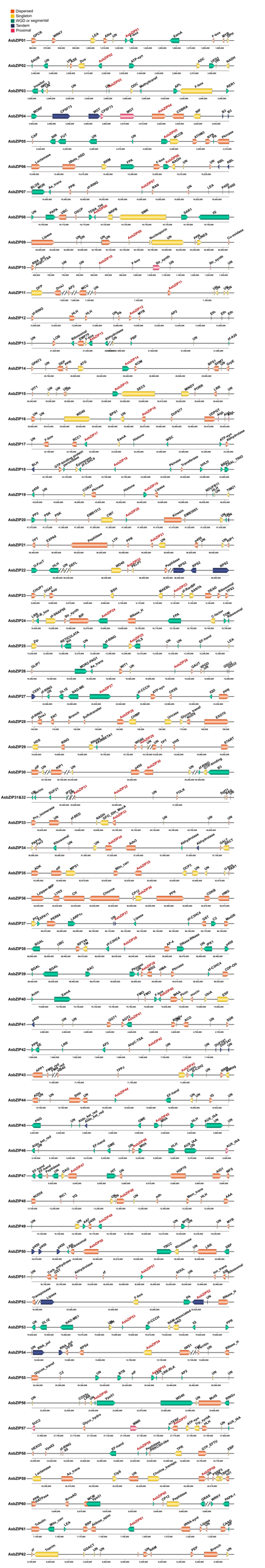

Supplement: Supplementary Figure 4 — Gene duplicated AsbZIPs and their nearby genes within gene clusters. [file DataSheet_4.pdf]
